# Latitudinal variations in morphometric traits and bioenergetic status of adult red squat lobsters *Grimothea monodon* (H. Milne Edwards, 1837) in the Southeast Pacific Ocean

**DOI:** 10.7717/peerj.20339

**Published:** 2025-11-17

**Authors:** Marco Quispe-Machaca, Maximiliano Zilleruelo, Pepe Espinoza, Gabriela Torres, Ángel Urzúa

**Affiliations:** 1 Facultad de Ciencias, Universidad Católica de la Santísima Concepción, Programa de Doctorado en Ciencias Mención Biodiversidad y Biorecursos, Concepción, Biobío, Chile; 2 Instituto de Fomento Pesquero (IFOP), Valparaíso, Valparaíso, Chile; 3 Facultad de Ciencias Biológicas y Veterinarias, Escuela de Biología Marina, Universidad Científica del Sur, Lima, Peru; 4 Instituto del Mar del Perú (IMARPE), Callao, Lima, Peru; 5 Alfred-Wegener-Institut für Polar und Meeresforschung, Helgoland, Schleswig-Holstein, Germany; 6 Departamento de Ecología, Facultad de Ciencias, Universidad Católica de la Santísima Concepción, Concepción, Biobío, Chile; 7 Centro de Investigación en Biodiversidad y Ambientes Sustentables (CIBAS), Universidad Católica de la Santísima Concepción, Concepción, Biobío, Chile

**Keywords:** Decapods, Lifestyles, Nutritional index, Latitudinal gradient, Environmental variability, Fishery management

## Abstract

Adults of the red squat lobster (*Grimothea monodon*) present two morphotypes (small-pelagic (SP) and large-benthic (LB)) in their wide geographic distribution range in the Southeastern Pacific Ocean (SEPO). In this marine ecosystem, they are exposed to conspicuous latitudinal variations in oceanographic and physicochemical parameters that affect their nutritional and fitness status. The objective of this study was to determine variations in the bioenergetic condition at the level of morphometric, sexual and biochemical traits of *G. monodon*, considering a wide spatial scale of their populations’ distribution along a latitudinal gradient (from 9°S to 36°S) in the SEPO. According to the environmental parameters, temperature and dissolved oxygen presented abrupt changes between 15°S–17°S, while chlorophyll and salinity showed a constant reduction along the latitudinal gradient. When environmental parameters were related to the size of the two morphotypes (SP, LB) of *G. monodon*, some trends of change were observed, while the relative condition factor showed significant differences along the latitudinal gradient. The biochemical condition of SP individuals showed an increasing trend in glucose from Chimbote to Chala, proteins showed abrupt changes in three zones (between Huacho-Lima, Lomitas, and Chala), and lipids showed a notable change between Lima-Cañete. In turn, in LB individual’s increases were recorded in all their biochemical constituents towards high latitudes. A slight variability in fatty acids was observed between SP individuals from the north (Chimbote, Huarmey, Huacho) and SP individuals from the south (Marcona, Chala, Planchada, Mollendo). In addition, significant latitudinal differences were observed in the fatty acids of the two morphotypes (SP, LP). The nutritional condition index (docosahexaenoic acid (DHA)/eicosapentaenoic acid (EPA) ratio) showed significant differences for the locality factor. Our findings revealed conspicuous differences in the bioenergetic condition of *G. monodon* adults at the latitudinal level. These variations were strongly linked to the predominant environmental conditions in the SEPO. It is consequently recommended that future sustainable exploitation models consider a physiological and ecosystemic approach that includes key aspects of the nutritional condition and its habitat, thus establishing, in real time, the health status of the natural populations of this resource.

## Introduction

Ectothermic invertebrates that have a wide distribution range in marine ecosystems are exposed to a high variability in environmental conditions and/or physicochemical parameters (temperature, salinity, oxygen, food availability) typical their natural habitat (Zeng et al., 2020). These changes spatially depending on the latitude and/or climatic zones and consequently influence the energy requirements and costs (trade-offs) of the animals that live there (Gravel, Couture & Cooke, 2010), ultimately impacting their growth and survival rates (Gravel, Couture & Cooke, 2010). Among ectothermic marine invertebrates, decapod crustaceans are considered one of the most successful taxonomic groups, successfully colonizing and inhabiting a wide variety of marine ecosystems (Benvenuto, Knott & Weeks, 2015; Behringer & Duermit-Moreau, 2021). In this context, some key traits of decapods (body size, sex, degree of reproductive maturity) have been observed to vary as a function of the latitudinal gradient (Steele, 1988; Colpo, Mulreedy & Negreiros-Fransozo, 2022). These variations in the sexual and morphometric parameters of decapods have been recognized as plastic and/or presumably adaptive responses to the latitudinal changes in ocean physicochemical parameters including temperature, dissolved oxygen, and salinity (Jaramillo et al., 2017; Zeng et al., 2020). Also, in decapods of importance in the fishing industry (*Homarus americanus*; *Cancer pagurus*; *Metacarcinus edwardsii*), it has been reported that, along with the variability in key oceanographic parameters, overfishing has also influenced their size at first reproductive maturity and the sex ratio of adult individuals (Moore et al., 2022; Hamame, 2023). Therefore, given the global increase in crustacean fisheries resulting from declining fish stocks (Smith & Addison, 2003; Wu et al., 2020; Boenish et al., 2021) with their contribution rising from 4% to 7% (Boenish et al., 2021), the sustainable exploitation of this resource is now more important than ever. Thus, biological, fishery and environmental aspects must be included in the development of management activities and fishing models under an ecosystemic approach.

Our model species, the red squat lobster *Grimothea monodon* (previously known as *Pleuroncodes monodon*) (Machordom et al., 2022), has a wide geographic distribution range in the Southeastern Pacific Ocean (SEPO), from the Lobos de Afuera Islands in Perú (5°S) to the Chiloe Islands (42°S) in Chile (Hendrickx & Harvey, 1999; Hernáez & Wehrtmann, 2014). Therefore, they are exposed to conspicuous latitudinal variations in oceanographic and physicochemical parameters throughout their geographic distribution range in the SEPO (Green et al., 2014). In this environmental context, a notable decrease in seawater temperature and salinity towards high latitudes has been described in the SEPO, as well as significant spatial changes in dissolved oxygen levels and nutrients (chlorophyll) (Silva, Mesquita & Paula, 2010; Díaz-Astudillo et al., 2024). Adults individuals of *G. monodon*, in the SEPO, present dual and distinctive body traits, including small pelagic (SP: Perú and northern Chile, 5°S–24°S) and large benthic (LB: Chile, ∼24°S to 48°S) individuals (Hendrickx & Harvey, 1999; Hernáez & Wehrtmann, 2011). Through genetic analysis, it was determined that both body types represent a single species due to its high dispersal potential and the connectivity between its populations (Haye et al., 2010). The pelagic morphotype is extracted along with other fishery resources, such as anchovy, and is also used in the biotechnology industry to obtain pigments (Gutiérrez et al., 2008; Barriga-Sánchez et al., 2023). Meanwhile, the benthic morphotype has been captured by industrial fishing fleets for several decades (Palma & Arana, 1997), which has led to overfishing in the Chilean LB population, generating temporary closures of extractive activities (*i.e.,* fishing bans), and sustainable fishery management plans based on a precautionary approach to annual catch quotas for this resource (SUBPESCA, 2023).

Decapod fishery management models, such as the age–size structured assessment of crustacean fisheries (see Smith & Addison, 2003; Punt, Huang & Maunder, 2013; Kilada & Driscoll, 2017; Hodgdon et al., 2022), must first establish the biomass stock of the resource. Current assessment models for crustaceans exploited in the HCE (IFOP, 2025) typically consider weight and size as separate variables rather than in combination. However, integrative reference indices—such as the relative condition factor (Kn) and biochemical markers (*e.g.*, glucose, proteins, lipids, fatty acids)—which capture both morphological traits and bioenergetic condition, can provide more comprehensive estimates of individual health status across latitudinal gradients. Thus, data on the relationship between body length (L) and weight (W) are collected (De Carvalho-Souza et al., 2023), allowing for the weight of an individual to be estimated based on its size (Pinheiro & Fiscarelli, 2009). However, this morphometric relationship (L-W) can vary between populations along a latitudinal gradient according to physiological factors that operate at the intra-individual level, such as sex, reproductive maturity (females with and without eggs) and growth rates (Jisr et al., 2018). Therefore, it is important to consider other fundamental parameters and/or indices, such as the Kn (based on relation length-weight; Jisr et al., 2018) and the bioenergetic condition (based on the energy reserves of the biochemical composition, see Couillard et al. (2023) and Guzmán-Rivas et al. (2021a)). Integrating these indices provides a holistic perspective that can more accurately reflect the health status of the exploited populations throughout a wide geographical area (Rodrigues et al., 2013; Guzmán-Rivas et al., 2021a; Guzmán-Rivas, Quispe & Urzúa, 2022).

From a bioenergetic perspective, Kn and energy reserves can also serve as moderate indicators of the nutritional condition of exploited populations. Nevertheless, such interpretations should be made with caution. While Kn may reflect food availability in the environment (Froese, 2006; Gelabert, Hurtado & Pérez, 2019), improvements in individual condition can also arise from reduced population density (Cifuentes et al., 2012). From a physiological standpoint, the L–W relationship was initially formulated at the individual level to describe growth and subsequently extended to populations (von Bertalanffy, 1957). More recently, Dynamic Energy Budget (DEB) theory provides a mechanistic framework for characterizing metabolic processes in organisms, specifically detailing the allocation of energy and nutrients to growth, reproduction, and maintenance (Temming & Herrmann, 2009).

The Kn index, widely and historically applied in studies on fishes (Pangle & Sutton, 2005; Jin et al., 2015; Mazumder et al., 2016; Khristenko & Kotovska, 2017; Jisr et al., 2018), and very little in decapod crustaceans (Khademzadeh & Haghi, 2017; Suryanti, Riza & Raza’i, 2018; Gelabert, Hurtado & Pérez, 2019), is used to estimate the degree of adaptation and growth of a population in its natural environment (LeCren, 1951; Froese, 2006; De Carvalho-Souza et al., 2023). Because this index is considered a non-invasive method, it offers a valuable advantage for studies of fishery resources. However, in decapods, its limitation lies in the inability to detect significant changes in nutritional status that occur during intermolt periods and growth (Lopeztegui-Castillo, 2021). Particularly, based on body biomass (weight) and the content of bioenergetic reserves (lipids, proteins, glucose, fatty and amino acids) that are conservatively stored in the respective organs (or body parts), the nutritional condition and/or health of individuals can be determined (Rodrigues et al., 2013; Guzmán-Rivas et al., 2021a).

In decapods, bioenergetic reserves are involved in various biological processes (metabolism, migration, growth, reproduction) (Lloret & Planes, 2003; Rodríguez-Viera et al., 2017; Zuloaga et al., 2023), which reveals a functional link between bioenergetic reserves and the condition factor (Zakzok et al., 2022; Wang et al., 2024; Yang et al., 2024). These condition indices arise from the assumption that individuals with greater body biomass (W) and size (CL) have the capacity to store greater biochemical reserves (mainly glucose, lipids, proteins) and essential fatty acids (docosahexaenoic acid (DHA), eicosapentaenoic acid (EPA)) in their bodies (Guzmán-Rivas et al., 2021a). As a consequence, they have demonstrated a better nutritional condition and/or health status (Mozsár et al., 2015; Gubiani et al., 2020), aiding them when faced with changes in environmental factors (temperature, salinity, oxygen, food availability), leading to greater survival and reproductive success (Karametsidis et al., 2023; Lopeztegui-Castillo, Olivera-Espinosa & Abitia-Cárdenas, 2023).

In turn, the bioenergetic condition of decapods may be influenced not only by intra-individual factors (molt, sex, reproductive status), but also by the physicochemical factors in their environment (temperature, salinity, oxygen), as well as the oceanographic conditions (upwellings) that vary spatially throughout the SEPO (Araújo & Lira, 2012; De Carvalho-Souza et al., 2023). Recent studies Guzmán-Rivas et al., 2021a; Guzmán-Rivas et al., 2021b) have revealed that the differences recorded in the nutritional condition of juvenile *G. monodon* individuals from two distant nursery locations (Coquimbo ∼29° S *vs.* Concepción ∼36°S) are correlated with physicochemical and oceanographic parameters (temperature, salinity, oxygen level, upwelling intensity, food availability) of seawater, which vary spatially along the latitudinal gradient in the SEPO (Graco et al., 2016; Igarza et al., 2019; Deville et al., 2020; Lopeztegui-Castillo, 2021). Fishery models of decapods with wide distribution ranges often rely solely on morphometric parameters to assess the condition of the bioresource (Lopeztegui-Castillo, 2021). However, the use of morphometric measurements alone as indicators of nutritional condition is highly questionable (Mozsár et al., 2015). Therefore, it is necessary to develop a more detailed analysis that includes other key parameters including bioenergetic condition, sex, and reproductive status. These parameters should be integrated with one another and linked to environmental factors that modulate the nutritional condition of ectothermic organisms throughout their distribution gradient (Lopeztegui-Castillo, 2021; Couillard et al., 2023). In this study, we hypothesized that the bioenergetic status of *G. monodon* varies with body size and sexual traits across its distribution range in the SEPO, similar to patterns reported in other crustacean species with respect to body form and/or reproductive condition (*e.g.*, *Aristeus antennatus, Parapenaeus longirostris, Nephrops norvegicus* (Rosa & Nunes, 2003); *Charybdis japonica* (Xing-Hong et al., 2014); *Sesarma boulengeri* (Sinha & Ahmed, 1978); *Parastacus defossus* (Buckup et al., 2008); *Grimothea monodon* (Guzmán-Rivas, Quispe & Urzúa, 2022)). We further hypothesized that these variations are associated with latitudinal gradients in environmental physicochemical factors. Therefore, the objective of this study was to evaluate bioenergetic condition in the red squat lobster by integrating morphometric, sexual, and biochemical traits across a broad spatial scale, encompassing 12 populations distributed between 9°S and 36°S in the SEPO.

Furthermore, this study expands current knowledge of the bioenergetics of *G. monodon* (Guzmán-Rivas et al., 2021b; Quispe-Machaca et al., 2024) across its life history, incorporating reproductive and sexual traits of the adult phase (males, females, and non-ovigerous females) as well as morphotypes (pelagic *vs.* benthic) that occur along a broad latitudinal gradient under varying environmental conditions in the SEPO.

## Materials & Methods

### Study area and environmental physicochemical factors

The study area chosen was the SEPO zone where *G. monodon* has a high biomass and is frequently caught by the fishing fleet. Therefore, biological-fishing monitoring of this species is carried out by the Peruvian Sea Institute (Chimbote-Mollendo; 09°S–17°S; respectively) and the Fisheries Development Institute of Chile (Coquimbo-Concepción; 30°S–36°S; respectively). This large study area includes twelve localities (see Table S1) within the latitudinal gradient (from 09°S to 36°S) of the SEPO. At each of the sample collection sites, measurements of the physicochemical parameters of seawater (temperature, dissolved oxygen, salinity, chlorophyll) were carried out using a CTD instrument (Carhuapoma et al., 2023). These measurements were then corroborated and compared through the website platforms Giovanni (https://giovanni.gsfc.nasa.gov): National Aeronautics and Space Administration (NASA), and by Copernicus (https://copernicus.eu): Earth Observation Program of the European Union. All these environmental data were used to compare and model their variability among locations along the latitudinal gradient in the SEPO (Table S2).

### Sample collection

**Figure 1:**
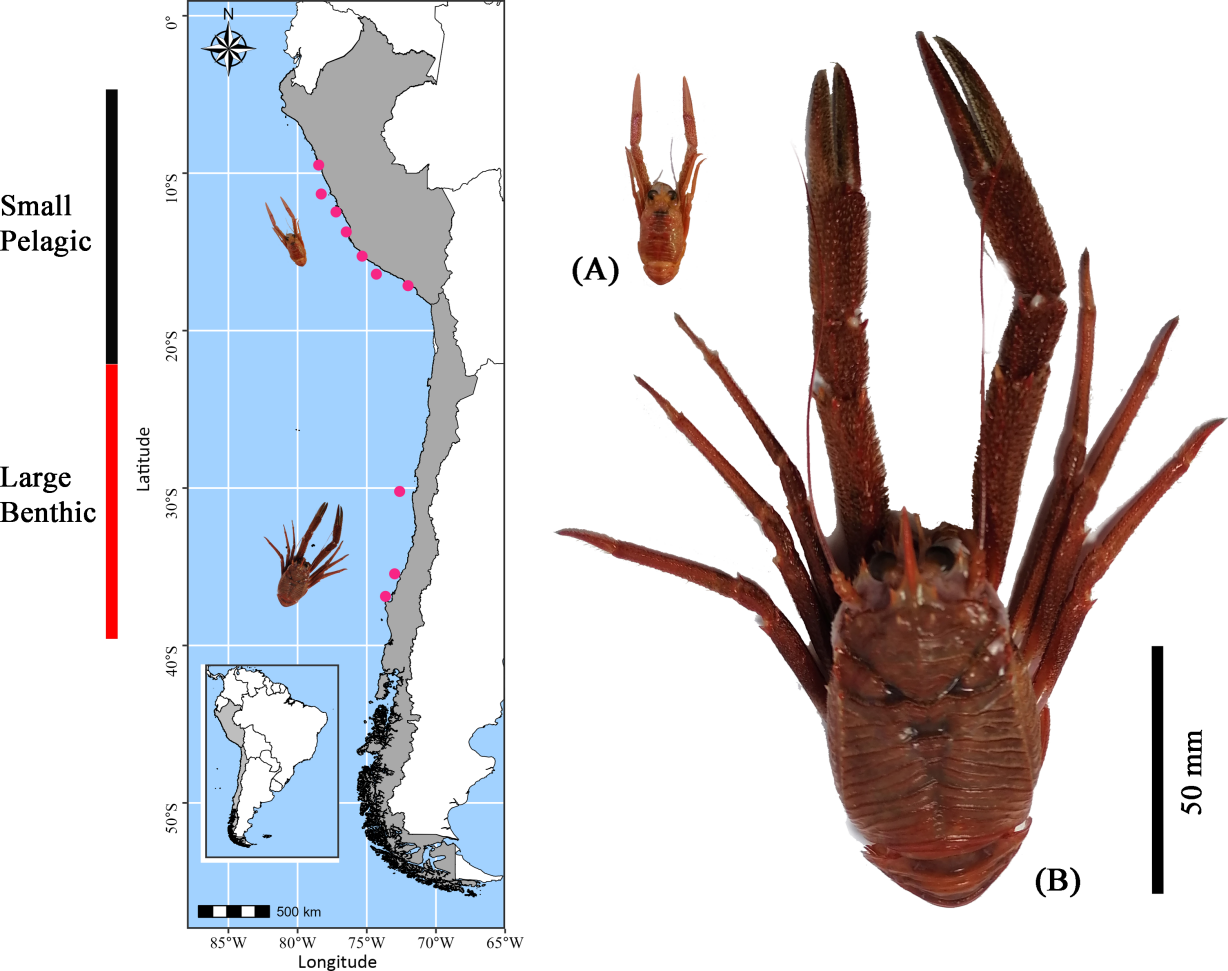
Sampling locations of *Grimothea monodon* in their two morphotypes: “small-pelagic (SP)” (Peru) and “large-benthic (LB)” (Chile) in the Southeastern Pacific Ocean.

Using the fishing fleet operating off the coast of Perú and Chile in the SEPO, adult red squat lobster individuals were collected in February (end of the austral summer) of 2022 at 12 distinct locations along the latitudinal gradient in the SEPO (from 9°S to 36°S) (for details see Table S1) (Fig. 1). Although *G. monodon* exhibits a prolonged reproductive period with three or more broods per year (Thiel et al., 2012; Yapur-Pancorvo et al., 2023), February was selected for specimen collection because benthic females begin reproduction after molting (Yapur-Pancorvo et al., 2023). In contrast, in the pelagic form, about 30% of females are ovigerous during this period (Franco-Meléndez, 2012), suggesting that energy reserves are simultaneously allocated to growth and reproduction (Quispe-Machaca et al., 2024). In these study areas, contrasting sizes and lifestyles of adult *G. monodon* individuals were recorded, as follows: “small-pelagic” (SP: Chimbote-Mollendo; 09°S–17°S) and “large-benthic” (LB: Coquimbo-Concepción; 30°S–36°S). During the collection events, individuals were kept cold (in airtight boxes with dry ice) and transported by plane to the laboratory to be frozen at −80 °C for later analysis. A total of *N* = 1, 352 specimens were considered for determinations of sexual and/or reproductive traits (male, non-ovigerous female, ovigerous female), morphometric (weight: W; cephalothorax length: CL; sex ratio; relative condition factor: Kn), see Table 1. In turn, for determinations of bioenergetic status (measured as the content of glucose, lipid, protein, and fatty acid profile) a total of *N* = 748 were analyzed (ranging from 4 to 12 individuals per sexual category, see Table 2). For details on the number of individuals analyzed for each sexual status in each sampling area, see Tables 1, 2.

**Table 1:** Parameters of the length-weight relationship and relative condition factor (Kn) of *Grimothea monodon* in their lifestyles: “small-pelagic (SP)” (09°S–17°S) and “large-benthic (LB)” (30°S–36°S).

**Morphotype**	**Locality**	**Sex**	**N**	**b**	**a**	***R*** ^**2**^	**Kn**
**Small Pelagic (Perú)**	**Chimbote** **(9°S)**	**Male**	49	2.262	0.001	0.42	1.05 ± 0.17
		**Non-ovigerous female**	45	1.9924	0.0022	0.503	1.01 ± 0.19
		**Ovigerous female**	27	2.8339	0.0002	0.635	0.95 ± 0.23
	**Huarmey** **(10°S)**	**Male**	76	3.7139	0.00001	0.33	1.48 ± 0.41
		**Non-ovigerous female**	28	1.4218	0.0131	0.472	1.02 ± 0.19
		**Ovigerous female**	80	1.5112	0.0092	0.311	1.01 ± 0.16
	**Huacho** **(11°S)**	**Male**	36	3.6325	0.00002	0.618	0.91 ± 0.23
		**Non-ovigerous female**	12	1.1936	0.0285	0.647	1.00 ± 0.07
		**Ovigerous female**	53	2.7959	0.0002	0.411	1.10 ± 0.22
	**Lima** **(12°S)**	**Male**	39	2.9845	0.0001	0.726	1.51 ± 0.25
		**Non-ovigerous female**	45	2.1142	0.0019	0.611	1.04 ± 0.17
		**Ovigerous female**	49	3.561	0.00003	0.655	0.92 ± 0.14
	**Cañete** **(13°S)**	**Male**	38	2.3414	0.0009	0.674	1.06 ± 0.12
		**Non-ovigerous female**	30	2.0137	0.0024	0.525	1.01 ± 0.16
		**Ovigerous female**	35	2.4472	0.0007	0.598	1.04 ± 0.16
	**Lomitas** **(14°S)**	**Male**	34	2.3964	0.0009	0.841	0.99 ± 0.11
		**Non-ovigerous female**	37	3.9827	0.000007	0.764	1.09 ± 0.22
		**Ovigerous female**	13	2.1785	0.0017	0.765	1.03 ± 0.13
	**Marcona** **(15°S)**	**Male**	39	2.7489	0.0003	0.636	1.02 ± 0.14
		**Non-ovigerous female**	62	3.1063	0.0001	0.721	0.98 ± 0.18
		**Ovigerous female**	39	2.8909	0.0002	0.737	1.05 ± 0.18
	**Chala** **(16°S)**	**Male**	52	3.5084	0.00003	0.796	1.03 ± 0.22
		**Non-ovigerous female**	36	4.1683	0.000003	0.843	1.20 ± 0.30
		**Ovigerous female**	47	4.3306	0.000003	0.849	1.01 ± 0.23
	**Planchada** **(16°34′S)**	**Male**	76	2.8289	0.0002	0.869	1.16 ± 0.14
		**Non-ovigerous female**	27	3.0195	0.0001	0.73	1.32 ± 0.20
		**Ovigerous female**	56	3.1892	0.00001	0.894	0.99 ± 0.11
	**Mollendo** **(17°)**	**Male**	63	2.7551	0.00002	0.558	1.20 ± 0.36
		**Non-ovigerous female**	67	1.5733	0.0048	0.304	1.02 ± 0.23
		**Ovigerous female**	15	2.3536	0.0007	0.818	1.01 ± 0.10
**Large Benthic (Chile)**	**Coquimbo** **(30°S)**	**Male**	18	2.3116	0.0021	0.24	1.04 ± 0.21
		**Non-ovigerous female**	NR	NR	NR	NR	NR
		**Ovigerous female**	8	2.1574	0.0024	0.54	1.00 ± 0.08
	**Concepción** **(36°S)**	**Male**	15	2.362	0.0012	0.716	0.96 ± 0.05
		**Non-ovigerous female**	NR	NR	NR	NR	NR
		**Ovigerous female**	14	5.4199	3.00E−09	0.692	0.90 ± 0.07

**Notes.**

N, Number of individuals; a & b, Regression coefficient parameters; *R*^2^, Correlation coefficient; NR, Not Recorded.

**Table 2:** Bioenergetic condition (glucose, proteins, lipids) expressed in dry weight (DW) of *Grimothea monodon* in their lifestyles: “small-pelagic (SP)” (09°S–17°S) and “large-benthic (LB)” (30°S–36°S).

**Locality**		**Glucose (mg 20 mg DW** ^−1^ **)** **$\bar {X}$ ± SD**						**Proteins (mg 20 mg DW** ^−1^ **)** **$\bar {X}$ ± SD**						**Lipids (mg 20 mg DW** ^−1^ **)** **$\bar {X}$ ± SD**					
		**N**	**Male**	**N**	**Non-ovigerous female**	**N**	**Ovigerous female**	**N**	**Male**	**N**	**Non-ovigerous female**	**N**	**Ovigerous female**	**N**	**Male**	**N**	**Non-ovigerous female**	**N**	**Ovigerous female**
**SP**	**Chimbote (9°S)**	12	0.24 ± 0.11	12	0.24 ± 0.07	12	0.30 ± 0.08	12	6.99 ± 1.52	12	8.62 ± 2.18	12	5.86 ± 1.14	12	1.30 ± 0.47	12	1.38 ± 0.27	12	2.12 ± 0.90
	**Huarmey (10°S)**	12	0.41 ± 0.15	12	0.49 ± 0.14	10	0.41 ± 0.06	12	7.70 ± 1.27	10	9.88 ± 1.12	12	9.95 ± 1.52	12	2.07 ± 0.49	12	2.13 ± 0.74	12	2.15 ± 0.40
	**Huacho (11°S)**	11	0.36 ± 0.13	12	0.43 ± 0.06	4	0.30 ± 0.10	11	10.67 ± 1.49	4	11.02 ± 1.35	12	9.64 ± 1.66	12	0.81 ± 0.23	6	1.39 ± 0.24	12	1.44 ± 0.91
	**Lima (12°S)**	12	0.62 ± 0.22	12	0.67 ± 0.14	12	0.54 ± 0.17	12	6.00 ± 1.19	12	7.59 ± 1.46	12	6.89 ± 1.54	12	0.41 ± 0.90	12	0.90 ± 0.58	12	0.76 ± 0.19
	**Cañete (13°S)**	12	0.63 ± 0.16	12	0.68 ± 0.22	12	0.60 ± 0.15	12	7.75 ± 2.49	12	7.63 ± 2.41	12	8.90 ± 2.28	12	0.38 ± 0.17	12	0.52 ± 0.27	12	0.98 ± 0.49
	**Lomitas (14°S)**	12	0.66 ± 0.20	6	0.56 ± 0.12	12	0.56 ± 0.16	12	8.36 ± 1.47	12	10.69 ± 1.76	6	10.08 ± 1.47	12	1.24 ± 0.36	12	1.69 ± 0.35	6	1.80 ± 0.45
	**Marcona (15°S)**	12	0.64 ± 0.14	12	0.83 ± 0.19	12	0.70 ± 0.13	12	4.50 ± 0.92	12	5.13 ± 1.61	12	4.36 ± 0.89	12	1.62 ± 0.22	12	1.92 ± 0.36	12	2.17 ± 0.56
	**Chala (16°S)**	11	0.83 ± 0.19	10	1.29 ± 0.24	9	1.09 ± 0.28	11	6.46 ± 1.96	9	7.01 ± 0.55	10	6.82 ± 0.84	12	1.22 ± 0.43	12	2.15 ± 0.61	12	1.75 ± 0.54
	**Planchada (16°34′S)**	12	0.61 ± 0.12	12	0.59 ± 0.14	11	0.70 ± 0.19	12	5.83 ± 0.83	11	5.54 ± 1.26	12	5.76 ± 0.62	12	1.80 ± 0.60	7	1.72 ± 0.67	12	1.55 ± 0.75
	**Mollendo (17°S)**	12	0.62 ± 0.11	NR	0.71 ± 0.15	12	NR	12	5.48 ± 0.96	12	4.18 ± 0.56	NR	NR	12	1.68 ± 0.43	8	2.06 ± 0.73	7	1.51 ± 0.40
**LB**	**Coquimbo (30°S)**	12	0.11 ± 0.03	NR	NR	9	0.44 ± 0.43	12	6.59 ± 1.01	NR	NR	9	6.37 ± 2.29	12	0.49 ± 0.10	NR	NR	9	0.93 ± 0.10
	**Concepción (36°S)**	12	0.43 ± 0.21	NR	NR	12	0.40 ± 0.08	12	6.15 ± 1.18	NR	NR	12	5.78 ± 1.63	12	0.84 ± 0.27	NR	NR	12	1.30 ± 0.11

**Notes.**

SP, Small Pelagic; LB, Large Benthic; $\bar {X}$, Mean; SD, Standard deviation; NR, Not Recorded.

### Sexual and morphometric traits, relative condition factor

For the determination of sexual traits, adult individuals in stage C of intermolt (Freeman et al., 1987; Promwikorn, Kirirat & Thaweethamsewee, 2004; Ghanawi & Saoud, 2012) were selected. These were visually classified according to the sexual dimorphism (males *vs.* females) apparent in their body structures, as follows: (i) observation of genital pore (Gutiérrez & Zuñiga, 1977), (ii) females without eggs (rounded pleon and absence of embryos under the abdomen), and (iii) ovigerous females (rounded pleon, modified pleopods, presence of embryos under the abdomen) (Espinoza-Fuenzalida et al., 2012; Thiel et al., 2012). For ovigerous females, only those with eggs in the initial stage of development (spherical eggs of a bright orange color and without an eyespot; Guzmán et al. (2020)) were selected. Subsequently, the morphometric parameters of the individuals (CL, W) were determined by using a vernier caliper (precision of 0.001 mm) and a digital balance model PT-124/35 (precision of 0.001 g), respectively (Gutiérrez & Zuñiga, 1977). With the data obtained from W and CL, a potential regression analysis was performed with the aim of obtaining the constants “a” and “b”, which are necessary for the calculation of the relative condition factor (kn), using the following formula: Kn = W/aCL^b^ (LeCren, 1951; Froese, 2006). In addition, the sex ratio, defined as Female/(Female + Male), was calculated for both morphotypes along the latitudinal gradient, where values approaching 1 indicate a high proportion of females (Franco-Meléndez, 2012).

### Bioenergetic status (glucose, proteins, lipids, fatty acids)

The bioenergetic status was based on measurements of the main biochemical constituents and/or energy fuel present in the bodies of the individuals (Clarke, Rodhouse & Gore, 1994; Sieiro, Otero & Aubourg, 2020). For this, the muscle tissue of the tail of the red squat lobster was extracted, which was freeze-dried at −80 °C for 48 h (FDU-7012, Operon). A standardized sample of 20 mg of dry weight of tail muscle tissue was used for each of the analyses (glucose, proteins, lipids, fatty acids). The determinations of these biochemical components were based on the same methodology used in previous studies and described in detail by Guzmán-Rivas et al. (2021a), Guzmán-Rivas et al. (2021b)). A brief description of the biochemical methods follows below.

#### Glucose

Glucose quantification was performed following the colorimetric method described by Tietz (1995) and adjusted by Guzmán-Rivas, Quispe & Urzúa (2022). The samples were homogenized with 500 uL of water, then a 10 uL aliquot of each sample was placed in an Eppendorf tube and the kit reagents were added. Subsequently, the samples, together with the standard glucose and a blank, were incubated (20 min) in a 96-well microplate and read in a BIOTECK spectrophotometer (ELx808) at a 490 nm wavelength. The glucose concentration was obtained through a division between the absorbance of the sample (corrected with the blank) and the absorbance of the standard glucose.

#### Proteins

For protein quantification, the colorimetric method described by Lowry et al. (1951) was used. The lyophilized samples were homogenized in 500 μL of ultrapure water. Five uL of each sample were extracted and placed in a 96-well microplate along with the prepared reagents of the protein kit. Then, the samples were incubated for 15 min at room temperature and read with the BIOTECK spectrophotometer (ELx808) at a 750 nm wavelength. Its quantification was obtained by a protein calibration curve based on different dilutions of the serum albumin standard (0.2 x/x−1.2 x/x).

#### Lipids

Lipid quantification was performed using the gravimetric method described by Folch, Lees & Sloane Stanley (1957) and modified by Cequier-Sánchez et al. (2008). The samples were placed in five mL of dichloromethane:methanol (2:1), and placed in an ultrasound bath (AC-120H equipment, MRC). Then, four mL of KCl (0.88%) was added and centrifuged (FASCIO TG1650-S) for 5 min at 1,500 RPM. The lower phase was extracted and dried with an injection of nitrogen gas (GLAS COL 109A YH-1). Subsequently, it was weighed on a digital balance (SARTORIUS LA230S) with a precision of 0.1 mg.

#### Fatty acids

The identification and quantification of fatty acids (FAs) measured as methyl esters (FAMEs) was performed using the methodology described by Urzúa & Anger (2011). A volume of one mL of lipids was used and esterified with two mL of sulfuric acid (1%) at 70 °C (Thermo-Shaker MRC model DBS-001) for 90 min. Subsequently, three washes with N-hexane (six mL-3 mL-3 mL) were performed, the upper phase was extracted and dried with nitrogen gas. FAMEs were measured in a gas chromatograph (GC: Agilent, model 7890A) with a DB 225 column (J and W Scientific: 30 m long, 0.25 internal diameter, and 0.25 mm film). Identification of FAMEs was performed by comparison with a marine-derived standard (Supelco 37 FAME mix 47885-U) and quantified using an internal response factor (23:0 FA incorporated prior to transmethylation) (Agilent ChemStation, Santa Clara, CA, USA) (Quispe-Machaca et al., 2021).

### Nutritional condition index

From the fatty acid profile, the contents of essential fatty acids (DHA: docosahexaenoic acid; EPA: eicosapentaenoic acid) in the muscle tissue of *G. monodon* were quantified to calculate the nutritional condition index (DHA/EPA) (Hu et al., 2017).

### Statistical analysis

Latitudinal variations of the physicochemical parameters (temperature, salinity, oxygen, chlorophyll) found in the study areas (08°S–37°S) were analyzed using a general additive model (Zuur, Ieno & Elphick, 2010). The sex ratio was evaluated using a Pearson chi-square (*χ*2) test to compare differences between morphotypes (SP *vs.* LB) (Franco-Meléndez, 2012). Considering the CL is highly different between two large groups (SP and LB), a two-way analysis of variance (2-way ANOVA; fixed factors: sexual status, location) was performed independently for each group. In turn, using an integrated and/or standardized measure of the weight of the individuals, the latitudinal variations in the Kn and bioenergetic status (glucose, proteins, lipids, FAs, DHA/EPA ratios) were tested using a 2-way ANOVA (fixed factors: sexual status, locations). For the multivariate analysis of the FA profiles, the percentage was used. For this, the data was first transformed to the fourth root and a Bray Curtis resemblance matrix was performed. Then, an exploratory analysis of principal coordinates (PCoA) was conducted; based on the similarity percentage (SIMPER), the FAs that contributed at least 5% to this differentiation were identified. Subsequently, a permutational multivariate analysis of variance (PERMANOVA) was performed together with a similarity analysis (ANOSIM), with similarity between groups (R ≈ 0) and highly dissimilar groups (R ≈1). All these analyses were developed through the R platform and the Primer v7 software with a significance level of <0.05 following the methods described by Zuur, Ieno & Elphick (2010) and Anderson, Gorley & Clarke (2008) and Anderson (2017).

## Results

### Physicochemical parameters (temperature, dissolved oxygen, salinity, chlorophyll) in the SEPO study areas

**Figure 2:**
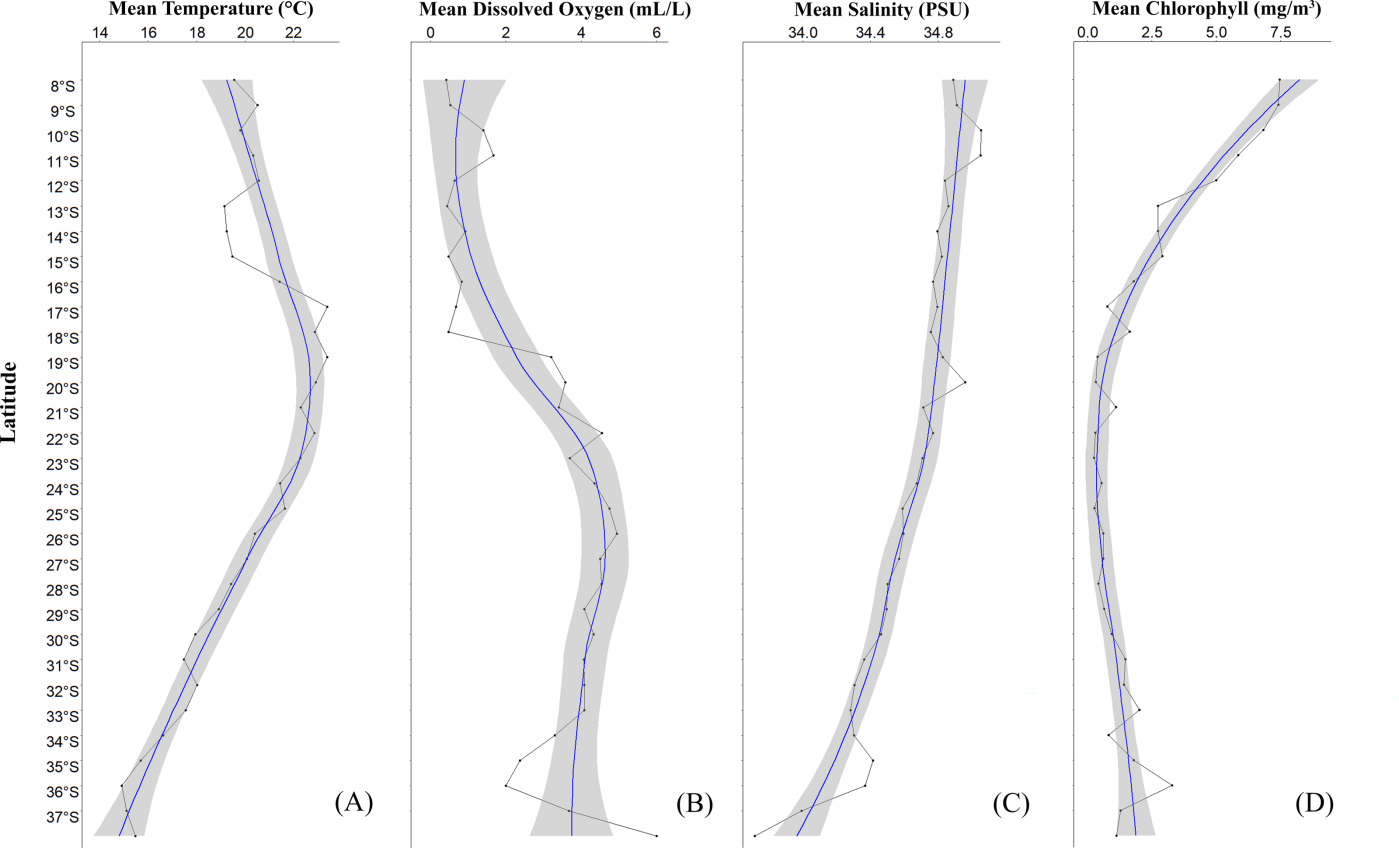
Average latitudinal variability of sea temperature (°C), dissolved oxygen (mL/L), salinity (PSU) and chlorophyll (mg/m3) between 08°S–37°S off the coasts of Peru and Chile, during the summer season. The solid line with dots (black color) corresponds to the average values at each latitudinal degree; the solid line (blue color) represents the estimated smoothed model; the gray band represents the 95% confidence interval.

A significant variability in the physicochemical parameters of seawater (temperature (^∘^C), dissolved oxygen (mL/L), salinity (PSU), chlorophyll (mg/m3)) along the latitudinal gradient between 8°S and 37°S was recorded (Fig. 2, see Table S2). Here, with respect to temperature, a reduction was observed from low to high latitudes (Fig. 2A). Particularly, an increase in temperature was recorded from 15°S to 17°S, and from there a continuous decrease in this parameter was detected. In the northern part of Perú, a consistently low temperature of 19.21 ± 1.77 °C was observed between 13°S and 15°S (Cañete-Marcona) and a maximum temperature of 22.41 ± 1.81 °C was recorded between 16°S- and 17°S (Chala-Mollendo). Regarding dissolved oxygen (Fig. 2B), three zones with high dissolved oxygen concentrations were observed: one in Perú and two in Chile. The first zone was off the coast of Perú between 10°S and 11°S (Huarmey-Huacho), with a volume of 1.54 ± 1.77 mL/L, the second was off the northern coast of Chile between 19°S and 21°S, with a volume of 3.40 ± 1.97 mL/L, and the third was off the central-southern coast of Chile between 25°S and 26°S, with a volume of 4.85 ± 1.03 mL/L of dissolved oxygen. In relation to salinity (Fig. 2C), a similar trend to temperature was observed, with the saline concentration decreasing from low to high latitudes (Fig. 2D). Similarly, three zones of slight peaks were observed: the first between 9°S and 11°S (Huarmey-Huacho) off the coast of Perú with a salinity of 34.96 ± 0.12 PSU, the second between 19°S and 20°S, with a salinity of 34.89 ± 0.13 PSU, and the third between 35°S and 36°S, with a salinity of 34.39 ± 0.22 PSU. Finally, chlorophyll also showed significant latitudinal variations, with high values between 09°S and 19°S and minimum values between 20°S and 28°S, and with a subsequent increase in chlorophyll towards 36°S (Concepción) (Fig. 2D).

### Morphometric (CL, W, b, Kn) and sexual traits (males, non-ovigerous females, ovigerous females)

**Figure 3:**
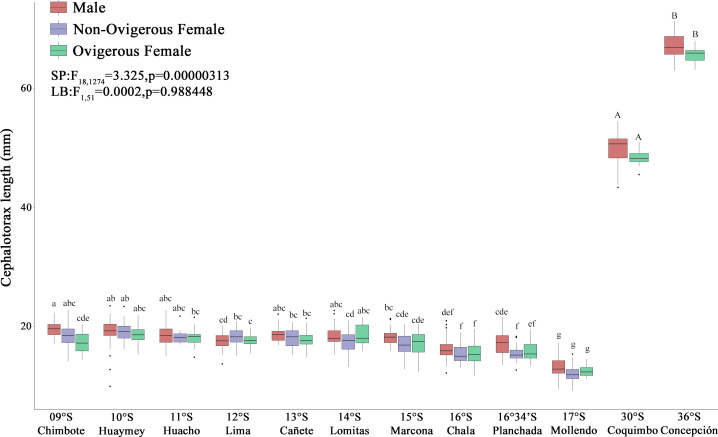
Size variability (cephalothorax length, CL) of *Grimothea monodon* in their lifestyles: “small-pelagic (SP)” (09°S–17°S) and “large-benthic (LB)” (30°S–36°S) in the Southeast Pacific Ocean. (i) different letters indicate significant differences within or between treatments, and (ii) the same letters indicate no significant differences within or between treatments.

The red squat lobster (*G. monodon*) presented a clear and significant spatial difference with respect to size between the traits “SP” (09°S–17°S) and “LB” (30°S–36°S) (Fig. 3). The sex ratio was 0.62 ± 0.06 in SP individuals, whereas LB individuals exhibited a sex ratio of 0.40 ± 0.12, with significant differences observed between morphotypes (*χ*2 = 9.342, g.l. = 1, *p* = 0.002). In turn, the average CL of the SP individuals in Perú was 19.03 ± 1.57 mm and the average W was 0.82 ± 0.20 g, while the LB individuals in Chile presented a CL of 49.44 ± 2.65 mm and a W of 15.99 ± 5.25 g (Fig. 3). According to Table 1, it can be observed that, depending on the sexual state, some groups of SP males, non-ovigerous females and ovigerous females presented a positive allometric growth (b > 3) or negative allometric growth (b < 3). While almost all groups of LB males, non-ovigerous females and ovigerous females, presented negative allometric growth, except for the LB ovigerous females from Concepción (36°S) (Table 1).

**Figure 4:**
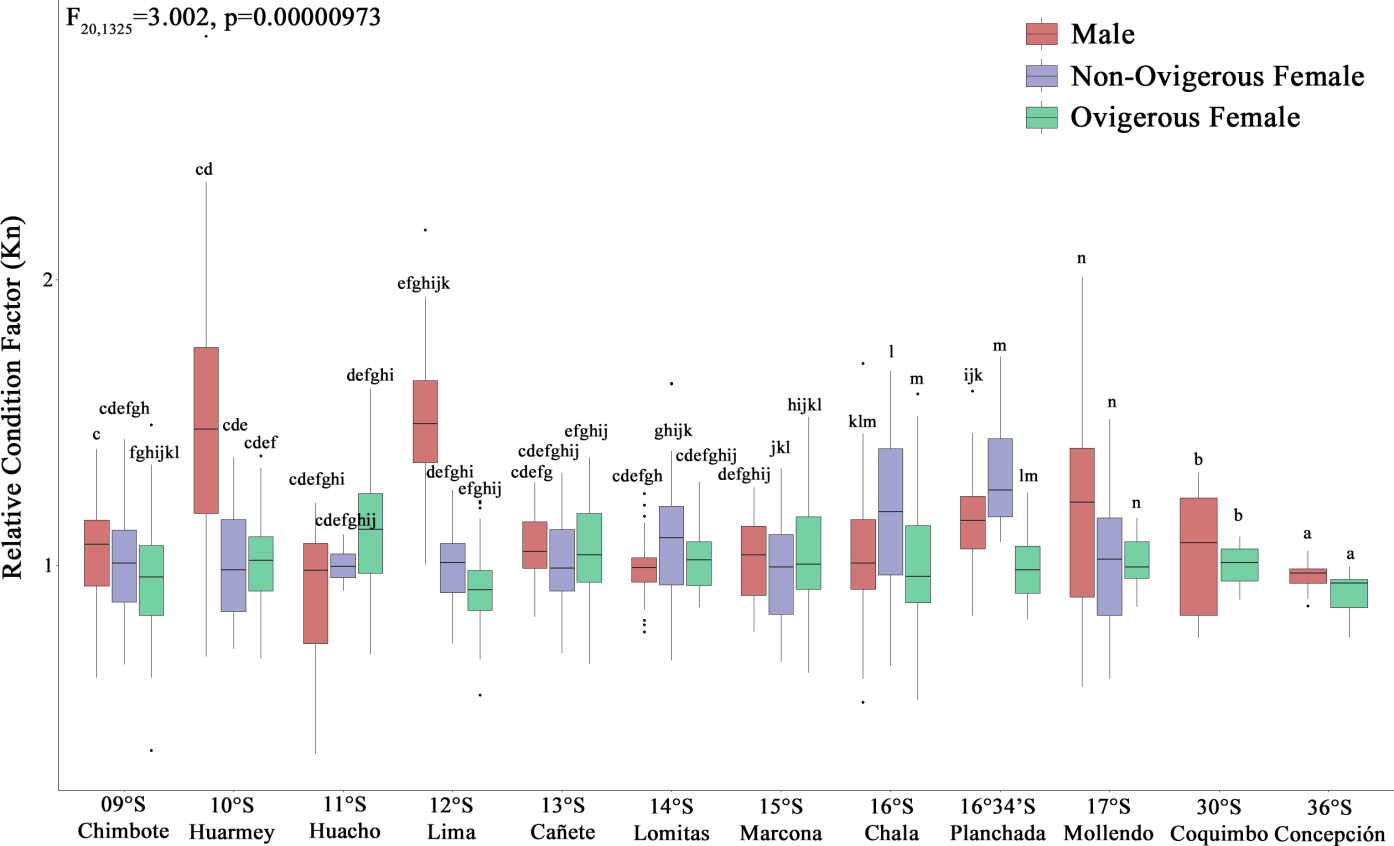
Relative condition factor (Kn) variability of *Grimothea monodon* in their lifestyles: “small-pelagic (SP)” (09°S–17°S) and “large-benthic (LB)” (30°S–36°S) in the Southeast Pacific Ocean. (i) different letters indicate significant differences within or between treatments, and (ii) the same letters indicate no significant differences within or between treatments.

According to the Kn, all analyzed individuals (males, non-ovigerous females, ovigerous females) with both body traits (SP and LB) presented an “optimal Kn”, corroborated by Kn values close to 1 (Fig. 4). Small pelagic (SP) males from the localities of Huarmey (10°S) and Lima (12°S) presented the best condition, followed by SP non-ovigerous females from the localities of Chala (16°S) and the Planchada (16°34′S). In turn, the condition of LB males and females tended to decrease slightly from Coquimbo (30°S) to Concepción (36°S). Significant variability in Kn was observed in some locations with respect to the sex factor. Thus, a 2-way ANOVA was carried out, which demonstrated significant statistical differences in the interaction “location*sexual status” (*F*_20,1325_ = 3.002, *p* < 0.05) (Fig. 4).

### Bioenergetic status (glucose, proteins, lipids, fatty acids)

#### Glucose

**Figure 5:**
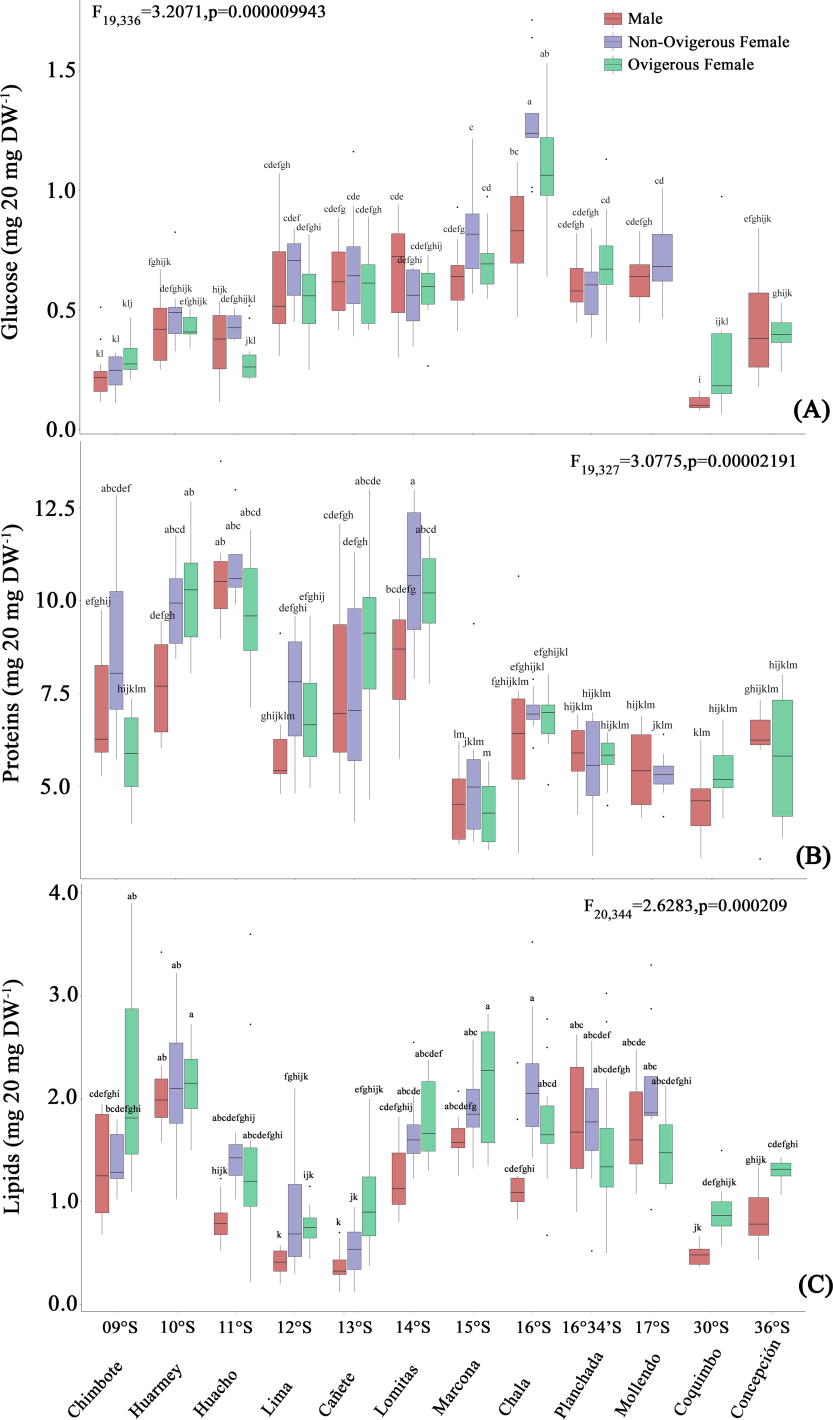
Biochemical composition variability (glucose (A), proteins (B), lipids (C)) of *Grimothea monodon* in their lifestyles: “small-pelagic (SP)” (09°S–17°S) and “large-benthic (LB)” (30°S–36°S). (i) different letters indicate significant differences within or between treatments, and (ii) the same letters indicate no significant differences within or between treatments.

The glucose content of *G. monodon*, at the sexual state level (male, non-ovigerous female, ovigerous female), revealed differences between localities along the latitudinal gradient (Fig. 5A, Table 2). In this case, the SP group showed an increase in their glucose concentration at all sexual states from Chimbote (9°S) to Chala (16°S), with individuals from the latter locality presenting the highest glucose content (Fig. 5A). Subsequently, a gradual reduction in the glucose content of individuals was observed, from Mollendo (17°S); minimum values were reached in low latitude localities (Chimbote-Huacho; 9°S–11°S). It is important to highlight that, when comparing the glucose content between groups and/or size categories (SP *vs* LB), the LB red squat lobsters presented a lower glucose content, and their values increased slightly from Coquimbo (30°S) to Concepción (36°S) in the SEPO. When comparing the glucose contents of the entire data pool along the latitudinal gradient, a 2-way ANOVA showed statistical differences, revealing a significant effect in the interaction between the factors: location*sex (*F*_19,336_ = 3.207, *p* < 0.05).

#### Proteins

The protein content of *G. monodon*, at the sex and locality levels, varied along the latitudinal gradient in the SEPO (Fig. 5B, Table 2). Here, an increase in the protein content of individuals was observed from low latitudes to high latitudes. Abrupt changes in protein content were also recorded in individuals from the localities of Huacho (11°S) and Lima (12°S), and later between Lomitas (14°S) and Chala (15°S). SP individuals from Marcona (15°S) to Mollendo (17°S), corresponding to the southern zone of Perú, presented similar values to those recorded in LB individuals in the Chilean localities. In these high latitude areas of the SEPO, an increase in the protein content was observed from 30°S (Coquimbo) to 36°S (Concepción). Considering the entire data set, the variations in the protein content of *G. monodon* observed at the level of the sex and location factors, showed a significant interaction of these factors along the latitudinal gradient in the SEPO (2-Way ANOVA; *F*_19,327_ = 3.0775, *p* < 0.05).

#### Lipids

As observed above with the other biochemical components, the lipid content of *G. monodon* adults also varied by sex and location in the SEPO (Fig. 5C, Table 2). SP individuals along the latitudinal gradient showed a decrease in their lipid contents from Chimbote (9°S) to Lima (12°S), then an increase in lipids was recorded from Cañete to Marcona (15°S), with a peak at 13°S (Fig. 5C, Table 2). Between the Chala (16°S) and Mollendo (17°S) locations, SP individuals consistently presented relatively similar lipid values. In turn, a low lipid content was observed in LB individuals compared to the different locations of the SP, except for Lima (12°S) and Cañete (13°S), which presented similar values to the locations of Coquimbo (30°S) and Concepcion (36°S). Also, an increase in lipids was recorded from the location of Coquimbo towards Concepción. The consistent variability observed in the lipid content of individuals along the latitudinal gradient in the SEPO was corroborated by a 2-way ANOVA, with a statistically significant interaction between the factors: location * sex (*F*_20,344_ = 2.6283, *p* < 0.05).

#### Fatty acids

A total of *N* = 25 different types of FAs were recorded in male, *N* = 21 in non-ovigerous female and *N* = 26 in ovigerous female individuals of *G. monodon* across the SEPO. Here, a clear difference in the total content of FAs (mg g DW^−1^) was observed between SP and LB individuals, with a lower FA content in LB compared to SP red squat lobster (Tables 3–5).

**Table 3:** Fatty acid profile in males of *Grimothea monodon* in their lifestyles: “small-pelagic (SP)” (09°S–17°S) and “large-benthic (LB)” (30°S–36°S).

**FAMES**	**Chimbote (9°S)**		**Huarmey (10°S)**		**Huacho (11°S)**		**Lima (12°S)**		**Cañete (13°S)**		**Lomitas (14°S)**		**Marcona (15°S)**		**Chala (16°S)**		**Planchada (16° 34′S)**		**Mollendo (17°S)**		**Coquimbo (33°S)**		**Concepción (36°S)**	
mgFA*gDW ^−1^	**$\bar {X}$ ± SD**	**%**	**$\bar {X}$ ± SD**	**%**	**$\bar {X}$ ± SD**	**%**	**$\bar {X}$ ± SD**	**%**	**$\bar {X}$ ± SD**	**%**	**$\bar {X}$ ± SD**	**%**	**$\bar {X}$ ± SD**	**%**	**$\bar {X}$ ± SD**	**%**	**$\bar {X}$ ± SD**	**%**	**$\bar {X}$ ± SD**	**%**	**$\bar {X}$ ± SD**	**%**	**$\bar {X}$ ± SD**	**%**
**C11:0**	ND	ND	ND	ND	ND	ND	0.25 ± 0.00	1.90	ND	ND	ND	ND	ND	ND	ND	ND	ND	ND	ND	ND	ND	ND	ND	ND
**C12:0**	ND	ND	0.47 ± 0.00	3.20	0.32 ± 0.00	2.61	0.33 ± 0.00	2.50	ND	ND	0.35 ± 0.00	3.42	0.37 ± 0.03	2.84	0.34 ± 0.00	2.78	0.47 ± 0.04	1.93	0.43 ± 00.8	1.63	ND	ND	ND	ND
**C13:0**	ND	ND	ND	ND	ND	ND	0.23 ± 0.03	1.76	ND	ND	ND	ND	ND	ND	ND	ND	ND	ND	ND	ND	ND	ND	ND	ND
**C14:0**	0.68 ± 0.56	9.35	0.67 ± 0.29	4.60	0.69 ± 0.42	5.57	0.66 ± 0.62	4.99	0.69 ± 0.35	9.14	0.52 ± 0.15	5.15	0.84 ± 0.51	6.42	0.77 ± 0.34	6.37	1.42 ± 0.38	5.87	1.91 ± 0.91	7.25	0.34 ± 0.11	4.29	0.30 ± 0.02	5.72
**C15:0**	0.62 ± 0.00	8.46	0.41 ± 0.08	2.84	0.25 ± 0.13	2.03	0.26 ± 0.04	1.98	0.28 ± 0.03	3.69	0.27 ± 0.09	2.63	0.35 ± 0.08	2.67	0.28 ± 0.04	2.34	0.38 ± 0.09	1.58	0.40 ± 0.08	1.51	0.19 ± 0.01	2.48	0.19 ± 0.01	3.67
**C16:0**	2.42 ± 1.37	33.3	4.55 ± 1.57	31.3	4.69 ± 2.32	38.0	1.60 ± 0.53	12.2	2.92 ± 1.11	38.6	3.41 ± 0.99	33.5	4.27 ± 1.30	32.6	4.65 ± 2.03	38.3	7.70 ± 3.18	31.9	7.91 ± 3.25	30.03	1.78 ± 0.44	22.72	1.51 ± 0.53	29.0
**C17:0**	ND	ND	0.42 ± 0.18	2.89	0.44 ± 0.26	3.59	0.43 ± 0.29	3.24	0.30 ± 0.05	3.98	0.27 ± 0.10	2.68	0.33 ± 0.11	2.53	0.35 ± 0.11	2.87	0.42 ± 0.10	1.75	0.44 ± 0.08	1.65	0.30 ± 0.04	3.78	0.33 ± 0.04	6.31
**C18:0**	0.76 ± 0.24	10.4	1.36 ± 0.33	9.37	1.36 ± 0.37	11.01	0.66 ± 0.24	4.97	0.73 ± 0.25	9.61	1.27 ± 0.34	12.5	1.36 ± 0.30	10.4	1.31 ± 0.36	10.8	2.33 ± 2.18	9.67	1.74 ± 1.76	6.60	0.65 ± 0.16	8.26	0.80 ± 0.13	15.4
**C20:0**	ND	ND	ND	ND	ND	ND	ND	ND	ND	ND	ND	ND	ND	ND	ND	ND	ND	ND	ND	ND	0.17 ± 0.01	2.13	0.17 ± 0.02	3.26
**C23:0**	ND	ND	ND	ND	ND	ND	ND	ND	ND	ND	ND	ND	ND	ND	ND	ND	0.92 ± 0.00	3.79	ND	ND	ND	ND	ND	ND
**Σ SFA**	4.47 ± 1.16	61.5	7.87 ± 1.78	54.2	7.75 ± 1.98	62.8	4.42 ± 0.64	33.5	4.92 ± 1.30 ±	65.0	6.08 ± 1.28	59.9	7.52 ± 1.57	57.4	7.70 ± 1.87	63.5	13.64 ± 3.26	56.5	12.83 ± 3.34	48.7	3.42 ± 0.62	43.7	3.30 ± 0.54	63.3
**C14:1**	ND	ND	ND	ND	ND	ND	0.28 ± 0.08	2.10	ND	ND	ND	ND	ND	ND	ND	ND	ND	ND	ND	ND	ND	ND	ND	ND
**C16:1**	0.89 ± 0.70	12.2	0.99 ± 0.58	6.82	0.89 ± 0.30	7.17	0.66 ± 0.27	5.03	0.51 ± 0.23	6.68	0.51 ± 0.19	4.98	0.79 ± 0.29	5.99	0.73 ± 0.70	6.01	1.12 ± 0.58	4.64	2.62 ± 1.17	9.95	0.23 ± 0.02	2.94	0.23 ± 0.02	4.44
**C17:1**	0.58 ± 0.29	7.94	0.55 ± 0.23	3.80	0.74 ± 0.50	6.03	0.26 ± 0.00	1.93	ND	ND	ND	ND	ND	ND	ND	ND	0.46 ± 0.00	1.92	2.19 ± 0.00	8.32	ND	ND	ND	ND
**C18:1n9**	1.33 ± 0.62	18.3	2.40 ± 0.99	16.5	1.89 ± 0.49	15.29	1.33 ± 0.42	10.1	1.21 ± 0.44	16.1	1.84 ± 0.71	18.14	2.70 ± 0.54	20.6	1.90 ± 1.02	15.7	3.12 ± 1.66	12.9	3.87 ± 1.98	14.70	1.14 ± 0.50	14.53	1.36 ± 0.27	26.1
**C20:1**	ND	ND	0.54 ± 0.11	3.74	0.46 ± 0.00	3.71	0.81 ± 0.73	6.12	ND	ND	ND	ND	ND	ND	ND	ND	1.56 ± 0.00	6.46	ND	ND	ND	ND	ND	ND
**C22:1n9**	ND	ND	0.43 ± 00	2.94	0.62 ± 0.00	4.98	0.61 ± 0.43	4.66	ND	ND	ND	ND	ND	ND	ND	ND	ND	ND	0.70 ± 0.00	2.65	0.31 ± 0.00	3.95	0.32 ± 0.02	6.16
**ΣMUFA**	2.8 ± 0.67	38.5	4.92 ± 1.03	33.9	4.59 ± 0.68	37.2	3.94 ± 0.52	29.9	1.72 ± 0.50	22.7	2.35 ± 0.86	23.1	3.49 ± 1.07	26.6	2.63 ± 1.04	21.7	6.26 ± 1.58	25.9	9.39 ± 1.73	35.6	1.68 ± 0.59	21.4	1.91 ± 0.60	36.7
**C18:2n6t**	ND	ND	ND	ND	ND	ND	ND	ND	ND	ND	ND	ND	ND	ND	ND	ND	0.71 ± 0.00	2.92	0.51 ± 0.16	1.92	ND	ND	ND	ND
**C18:2n6c**	ND	ND	0.40 ± 0.01	2.77	ND	ND	0.51 ± 0.48	3.85	ND	ND	0.40 ± 0.00	3.91	0.33 ± 0.00	2.50	ND	ND	ND	ND	ND	ND	0.25 ± 0.04	3.23	0.27 ± 0.02	5.17
**C18:3n6**	ND	ND	ND	ND	ND	ND	ND	ND	ND	ND	ND	ND	ND	ND	0.38 ± 0.00	3.11	ND	ND	ND	ND	0.25 ± 0.01	3.20	0.25 ± 0.004	4.84
**C20:2**	ND	ND	ND	ND	ND	ND	0.29 ± 0.00	2.20	ND	ND	ND	ND	ND	ND	ND	ND	ND	ND	ND	ND	ND	ND	D	ND
**ΣPUFAn6**	ND	ND	0.40 ± 0.01	2.77	ND	ND	0.80 ± 0.42	6.05	ND	ND	0.40 ± 0.00	3.91	0.33 ± 0.00	2.50	0.38 ± 0.00	3.11	0.71 ± 0.00	2.92	0.51 ± 0.16	1.92	0.50 ± 0.03	6.43	0.52 ± 0.02	10.0
**C18:3n3**	ND	ND	ND	ND	ND	ND	0.82 ± 0.00	6.18	ND	ND	ND	ND	ND	ND	ND	ND	ND	ND	ND	ND	0.56 ± 0.09	7.16	0.51 ± 0.02	9.73
**C20:3n3**	ND	ND	ND	ND	ND	ND	2.12 ± 0.00	16.1	ND	ND	ND	ND	ND	ND	ND	ND	ND	ND	ND	ND	0.41 ± 0.08	5.24	0.35 ± 0.09	6.78
**C20:5n3**	ND	ND	0.62 ± 0.14	4.24	ND	ND	0.56 ± 0.24	4.25	0.53 ± 0.13	7.03	0.73 ± 0.29	7.16	0.94 ± 0.36	7.18	0.88 ± 0.00	7.26	1.71 ± 1.45	7.09	2.15 ± 1.24	8.15	0.71 ± 0.33	9.02	1.25 ± 0.66	24.0
**C22:6n3**	ND	ND	0.71 ± 0.36	4.91	ND	ND	0.53 ± 0.16	4.00	0.39 ± 0.27	5.21	0.60 ± 0.31	5.93	0.82 ± 0.40	6.25	0.54 ± 0.16	4.48	1.82 ± 1.85	7.55	1.49 ± 1.09	5.64	0.56 ± 0.27	7.08	0.95 ± 0.47	18.3
**ΣPUFAn3**	ND	ND	1.33 ± 0.24	9.14	ND	ND	4.02 ± 0.46	30.5	0.93 ± 0.22	12.2	1.31 ± 0.30	13.1	1.76 ± 0.36	13.4	1.42 ± 0.19	11.7	3.53 ± 1.60	14.6	3.63 ± 1.18	13.8	2.23 ± 0.26	28.5	3.07 ± 0.58	58.8
**ΣPUFA**	ND	ND	1.73 ± 0.23	11.9	ND	ND	4.82 ± 0.45	36.6	0.93 ± 0.22	12.2	1.73 ± 0.29	17.0	2.09 ± 0.39	15.9	1.80 ± 0.19	14.8	4.24 ± 1.56	17.6	4.14 ± 1.18	15.7	2.74 ± 0.26	34.9	3.59 ± 0.56	68.8
**Σ Fatty Acid**	7.27 ± 0.98	100	14.52 ± 1.46	100	12.34 ± 1.36	100	13.18 ± 0.57	100	7.57 ± 1.09	100	10.16 ± 1.11	100	13.10 ± 1.34	100	12.13 ± 1.62	100	24.14 ± 2.94	100	26.35 ± 2.26	100	7.84 ± 0.52	100	5.22 ± 0.56	100

**Notes.**

$\bar {X}$, Mean; SD, Standard Deviation; %, Percentage; SFA, Saturated Fatty Acid; MUFA, Monounsaturated Fatty Acid; PUFA, Polyunsaturated Fatty Acid; ND, Not Detected.

In SP individuals, males presented the highest variety in FAs at the Lima locality (12°S), where 20 types of FAs were identified, corresponding to 13.18 ± 0.57 mg*g DW^−1^, while the locality with the least variety in FAs was Chimbote (09°S), with a total of seven types of FAs, with average values of 7.27 ± 0.98 mg*g DW^−1^. In addition, in some localities of the Peruvian SEPO, trace amounts and/or the absence of polyunsaturated fatty acids (PUFAs) (Chimbote, Huacho; 09°S, 11°S) and partial PUFAs (Cañete, 13°S) were recorded. In turn, in SP non-ovigerous females from the Huarmey locality (10°S), a high variety of FAs (17 types) with a content of 20.67 ± 2.42 mg*g DW^−1^ was found, while the locality with the least variety of FAs was Huacho (11°S), with a total of 10 FAs that represented average values of 12.69 ± 1.82 mg*g DW^−1^. The only locality that registered trace amounts and/or the absence of PUFAs in SP non-ovigerous females was the Huacho locality (11°S). Regarding SP ovigerous females, the greatest variety of FAs was found in the Huarmey locality (10°S), with 18 types of FAs, representing a total content of 21.90 ± 1.89 mg*g DW^−1^. While the locality with the least variety of FAs, was the locality of Mollendo (17°S), where 11 FAs were recorded, with average values of 22.99 ± 3.16 mg*g DW^−1^.

**Table 4:** Fatty acid profile in non-ovigerous females of *Grimothea monodon* in the lifestyles: “small-pelagic (SP)” (09°S–17°S) captured in the Southeast Pacific Ocean.

**FAMES**	**Chimbote (9°S)**		**Huarmey (10°S)**		**Huacho (11°S)**		**Lima (12°S)**		**Cañete (13°S)**		**Lomitas (14°S)**		**Marcona (15°S)**		**Chala (16°S)**		**Planchada (16°S)**		**Mollendo (17°S)**	
mgFA*gDW ^−1^	**$\bar {X}$ ± SD**	**%**	**$\bar {X}$ ± SD**	**%**	**$\bar {X}$ ± SD**	**%**	**$\bar {X}$ ± SD**	**%**	**$\bar {X}$ ± SD**	**%**	**$\bar {X}$ ± SD**	**%**	**$\bar {X}$ ± SD**	**%**	**$\bar {X}$ ± SD**	**%**	**$\bar {X}$ ± SD**	**%**	**$\bar {X}$ ± SD**	
**C12:0**	0.37 ± 0.03	1.59	0.49 ± 0.07	2.35	0.42 ± 0.00	3.31	0.35 ± 0.03	2.10	0.35 ± 0.02	2.0	0.37 ± 0.05	2.37	0.30 ± 0.13	1.28	0.41 ± 0.00	1.70	0.46 ± 0.06	1.60	0.42 ± 0.00	1.58
**C13:0**	ND	ND	0.27 ± 0.00	1.29	ND	ND	ND	ND	ND	ND	ND	ND	0.21 ± 0.00	0.92	ND	ND	ND	ND	ND	ND
**C14:0**	1.47 ± 1.50	6.36	1.08 ± 0.79	5.20	0.99 ± 0.56	7.80	1.18 ± 1.04	7.20	1.74 ± 1.23	9.70	1.17 ± 0.51 ±	7.46	1.87 ± 0.81	8.05	1.68 ± 0.78	6.90	1.96 ± 0.68	6.83	2.37 ± 1.02	8.85
**C15:0**	0.57 ± 0.44	2.48	0.37 ± 0.08	1.78	0.35 ± 0.13	2.79	0.31 ± 0.08	1.90	0.29 ± 0.05	1.60	0.28 ± 0.05	1.81	0.35 ± 0.11	1.53	0.43 ± 0.18	1.80	0.40 ± 0.10	1.41	0.41 ± 0.10	1.52
**C16:0**	7.16 ± 6.99	30.9	5.04 ± 4.34	24.40	5.57 ± 2.06	43.90	4.84 ± 4.51	30.00	9.65 ± 4.47	39.0	5.86 ± 2.04	37.30	9.52 ± 3.29	41.10	8.88 ± 3.59	36.00	11.1 ± 3.70	38.70	10.7 ± 4.38	40.1
**C17:0**	0.34 ± 0.06	1.46	0.39 ± 0.10	1.90	0.40 ± 0.13	3.17	0.34 ± 0.18	2.10	0.25 ± 0.03	1.40	0.29 ± 0.05	1.87	0.38 ± 0.07	1.66	0.46 ± 0.17	1.90	0.48 ± 0.11	1.67	0.45 ± 0.15	1.69
**C18:0**	1.42 ± 1.02	6.13	1.30 ± 0.93	6.30	1.53 ± 0.34	12.10	1.02 ± 0.65	6.30	1.23 ± 0.56	6.90	1.50 ± 0.29	9.55	1.91 ± 0.44	8.23	1.83 ± 0.47	7.50	2.13 ± 0.58	7.41	1.56 ± 0.54	5.84
**C20:0**	0.28 ± 0.00	1.20	ND	ND	0.38 ± 0.00	3.01	0.21 ± 0.00	1.30	ND	ND	ND	ND	ND	ND	ND	ND	ND	ND	ND	ND
**C22:0**	ND	ND	0.74 ± 0.00	3.59	ND	ND	ND	ND	ND	ND	ND	ND	ND	ND	ND	ND	ND	ND	ND	ND
**C23:0**	ND	ND	1.64 ± 0.00	7.91	ND	ND	ND	ND	ND	ND	ND	ND	ND	ND	ND	ND	ND	ND	ND	ND
**ΣSFA**	11.6 ± 4.40	50.2	11.3 ± 2.64	54.7	9.65 ± 2.24	76	8.25 ± 2.77	51.0	10.8 ± 3.18	61	9.48 ± 2.27	60.3	14.6 ± 3.55	62.8	13.7 ± 3.47	56.0	16.6 ± 4.22	57.6	15.9 ± 4.41	59.5
**C16:1**	2.97 ± 3.60	12.83	1.82 ± 1.89	8.79	0.71 ± 0.31	5.62	1.46 ± 1.33	8.90	1.61 ± 3.18	9.0	1.17 ± 0.56	7.46	1.79 ± 0.82	7.73	1.77 ± 0.00	7.20	2.40 ± 1.21	8.35	2.76 ± 1.14	10.3
**C17:1**	1.55 ± 1.30	6.69	1.11 ± 0.84	5.34	0.64 ± 0.39	5.05	ND	ND	ND	ND	ND	ND	ND	ND	ND	ND	ND	ND	ND	ND
**C18:1n9**	3.91 ± 4.40	16.88	3.42 ± 3.33	16.5	1.69 ± 0.38	13.3	2.77 ± 2.68	17.0	3.14 ± 2.34	18.0	3.02 ± 1.24	19.2	4.78 ± 1.63	20.6	4.28 ± 0.00	17.0	5.45 ± 2.02	19.0	5.03 ± 1.90	18.8
**C20:1**	0.85 ± 0.00	3.67	0.65 ± 0.15	3.16	ND	ND	1.14 ± 0.00	7.0	0.86 ± 0.00	4.8	ND	ND	ND	ND	1.09 ± 0.00	4.40	ND	ND	0.95 ± 0.00	3.54
**ΣMUFA**	9.27 ± 3.41	40.1	6.99 ± 2.47	33.8	3.05 ± 0.60	24	5.36 ± 2.18	33.0	5.61 ± 2.04	31	4.20 ± 1.33	26.7	6.57 ± 1.98	28.4	7.14 ± 2.00	29.0	7.85 ± 2.25	27.3	8.74 ± 1.99	32.7
**C18:2n6t**	ND	ND	ND	ND	ND	ND	ND	ND	ND	ND	ND	ND	ND	ND	0.60 ± 0.01	2.50	0.48 ± 0.00	1.65	ND	ND
**C18:2n6c**	0.48 ± 0.05	2.07	0.57 ± 0.16	2.77	ND	ND	0.67 ± 0.00	4.1	0.32 ± 0.00	1.8	0.48 ± 0.00	3.02	0.52 ± 0.00	2.23	0.85 ± 0.00	3.5	ND	ND	0.24 ± 0.00	0.88
**C18:3n6**	0.36 ± 0.00	1.56	0.45 ± 0.19	2.19	ND	ND	0.58 ± 0.00	3.6	ND	ND	ND	ND	ND	ND	ND	ND	ND	ND	ND	ND
**ΣPUFA n6**	0.84 ± 0.08	3.63	1.03 ± 0.16	4.96	ND	ND	1.26 ± 0.06	7.7	0.32 ± 0.00	1.8	0.48 ± 0.00	3.02	0.52 ± 0.00	2.23	1.45 ± 0.14	5.9	0.48 ± 0.00	1.65	0.24 ± 0.00	0.88
**C18:3n3**	ND	ND	ND	ND	ND	ND	ND	ND	ND	ND	ND	ND	ND	ND	ND	ND	ND	ND	ND	ND
**C20:3n3**	0.64 ± 0.00	2.78	ND	ND	ND	ND	ND	ND	ND	ND	ND	ND	ND	ND	ND	ND	ND	ND	ND	ND
**C20:5n3**	0.78 ± 0.18	3.37	0.99 ± 0.77	4.78	ND	ND	0.84 ± 0.30	5.2	0.61 ± 0.17	3.4	0.91 ± 0.33	5.78	0.86 ± 0.36	3.72	1.27 ± 0.57	5.2	1.75 ± 0.58	6.08	1.02 ± 0.18	3.82
**C22:6n3**	ND	ND	0.36 ± 0.00	1.74	ND	ND	0.62 ± 0.14	3.80	0.48 ± 0.13	2.70	0.66 ± 0.26	4.20	0.68 ± 0.22	2.91	0.98 ± 0.49	4.00	2.12 ± 0.75	7.38	0.83 ± 0.15	3.11
**ΣPUFA n3**	1.42 ± 0.15	6.14	1.35 ± 0.73	6.52	ND	ND	1.46 ± 0.25	9.00	1.10 ± 0.16	6.20	1.57 ± 0.32	9.98	1.54 ± 0.30	6.63	2.25 ± 0.53	9.20	3.87 ± 0.77	13.5	1.86 ± 0.18	6.93
**ΣPUFA**	2.26 ± 0.20	9.77	2.37 ± 0.50	11.5	ND	ND	2.72 ± 0.22	17.00	1.42 ± 0.16	7.90	2.04 ± 0.32	13.0	2.05 ± 0.30	8.86	3.69 ± 0.51	15.00	4.35 ± 0.77	15.1	2.09 ± 0.25	7.81
**Σ Fatty Acid**	23.1 ± 3.86	100	20.7 ± 2.42	100	12.7 ± 1.82	100	16.3 ± 2.48	100	17.8 ± 2.89	100	15.7 ± 1.88	100	23.2 ± 3.35	100	24.5 ± 2.92	100	28.8 ± 3.67	100	26.8 ± 3.54	100

**Notes.**

$\bar {X}$, Mean; SD, Standard Deviation; %, Percentage; SFA, Saturated Fatty Acid; MUFA, Monounsaturated Fatty Acid; PUFA, Polyunsaturated Fatty Acid; ND, Not Detected.

**Table 5:** Fatty acid profile in ovigerous females of *Grimothea monodon* in their lifestyles: “small- pelagic (SP)” (09°S–17°S) and “large-benthic (LB)” (30°S–36°S).

**FAMES**	**Chimbote (9°S)**		**Huarmey (10°S)**		**Huacho (11°S)**		**Lima (12°S)**		**Cañete (13°S)**		**Lomitas (14°S)**		**Marcona (15°S)**		**Chala (16°S)**		**Planchada (16° 34′S)**		**Mollendo (17°S)**		**Coquimbo (33°S)**		**Concepción (36°S)**	
mgFA*gDW ^−1^	**$\bar {X}$ ± SD**	**%**	**$\bar {X}$ ± SD**	**%**	**$\bar {X}$ ± SD**	**%**	**$\bar {X}$ ± SD**	**%**	**$\bar {X}$ ± SD**	**%**	**$\bar {X}$ ± SD**	**%**	**$\bar {X}$ ± SD**	**%**	**$\bar {X}$ ± SD**	**%**	**$\bar {X}$ ± SD**	**%**	**$\bar {X}$ ± SD**	**%**	**$\bar {X}$ ± SD**	**%**	**$\bar {X}$ ± SD**	**%**
**C12:0**	ND	ND	0.35 ± 0.02	1.59	0.39 ± 0.01	1.84	0.37 ± 0.05	3.58	0.39 ± 0.02	2.23	0.35 ± 0.01	2.05	0.38 ± 0.05	1.39	0.43 ± 0.08	2.06	0.47 ± 0.04	1.63	ND		ND	ND	ND	ND
**C13:0**	ND	ND	ND	ND	ND	ND	0.25 ± 0.00	2.35	ND	ND	ND	ND	ND	ND	ND	ND	ND	ND	ND	ND	ND	ND	ND	ND
**C14:0**	1.49 ± 1.02	7.60	0.91 ± 0.42	4.17	1.29 ± 1.46	6.05	0.79 ± 0.48	7.62	1.49 ± 0.99	8.44	1.20 ± 0.67	7.09	2.08 ± 1.02	7.64	1.26 ± 0.56	5.99	2.36 ± 1.48	8.21	1.91 ± 0.83	8.32	0.32 ± 0.03	5.19	0.34 ± 0.13	3.37
**C15:0**	0.24 ± 0.14	1.21	0.34 ± 0.13	1.55	0.41 ± 0.12	1.94	0.26 ± 0.04	2.51	0.32 ± 0.08	1.79	0.34 ± 0.10	1.98	0.36 ± 0.06	1.33	0.43 ± 0.09	2.07	0.45 ± 0.16	1.56	0.43 ± 0.09	1.86	0.20 ± 0.03	3.24	0.18 ± 0.01	1.82
**C16:0**	7.08 ± 4.42	36.2	5.98 ± 1.98	27.3	7.24 ± 6.25	34.0	3.11 ± 2.10	29.9	6.49 ± 4.67	36.8	5.99 ± 2.88	35.3	10.6 ± 4.55	39.0	6.65 ± 2.60	31.6	10.2 ± 6.31	35.6	8.76 ± 3.93	38.1	1.72 ± 0.19	28.2	1.78 ± 0.29	17.7
**C17:0**	0.34 ± 0.16	1.76	0.28 ± 0.08	1.30	0.43 ± 0.15	2.02	0.28 ± 0.07	2.68	0.33 ± 0.05	1.87	0.38 ± 0.13	2.26	0.45 ± 0.07	1.66	0.42 ± 0.11	1.98	0.51 ± 0.13	1.77	0.34 ± 0.09	1.47	0.28 ± 0.02	4.58	0.28 ± 0.03	2.84
**C18:0**	1.27 ± 0.54	6.50	1.69 ± 0.36	7.73	1.71 ± 0.87	8.02	0.78 ± 0.36	7.52	1.15 ± 0.66	6.51	1.43 ± 0.69	8.44	2.01 ± 0.62	7.41	1.52 ± 0.31	7.23	1.81 ± 0.99	6.29	1.33 ± 0.40	5.76	0.68 ± 0.09	11.4	0.77 ± 0.12	7.66
**C20:0**	0.30 ± 0.00	1.52	0.24 ± 0.00	1.11	0.23 ± 0.00	1.07	ND	ND	ND	ND	0.14 ± 0.00	0.84	0.33 ± 0.00	1.20	ND	ND	ND	ND	ND	ND	0.16 ± 0.01	2.70	0.17 ± 0.02	1.66
**C22:0**	ND	ND	ND	ND	0.86 ± 0.00	4.04	ND	ND	ND	ND	ND	ND	ND	ND	ND	ND	ND	ND	ND	ND	ND	ND	0.38 ± 0.00	3.76
**C24:0**	ND	ND	ND	ND	ND	ND	ND	ND	0.33 ± 0.00	1.88	ND	ND	ND	ND	ND	ND	ND	ND	ND	ND	ND	ND	ND	ND
**ΣSFA**	10.7 ± 3.53	54.8	9.80 ± 2.33	44.8	12.6 ± 3.76	58.9	5.85 ± 1.45	56.1	10.5 ± 3.19	59.6	9.84 ± 2.41	57.9	16.2 ± 4.25	59.7	10.7 ± 2.58	50.9	15.8 ± 4.76	55.0	12.8 ± 3.72	55.5	3.35 ± 0.57	55.1	3.90 ± 0.60	38.8
**C14:1**	ND	ND	ND	ND	0.35 ± 0.00	1.64	ND	ND	ND	ND	ND	ND	ND	ND	ND	ND	ND	ND	ND	ND	ND	ND	ND	ND
**C16:1**	2.28 ± 1.76	11.7	1.67 ± 0.85	7.61	1.66 ± 1.84	7.81	0.98 ± 0.58	9.35	1.51 ± 1.24	8.58	1.21 ± 0.97	7.14	1.93 ± 1.23	7.10	1.86 ± 0.83	8.87	2.38 ± 1.65	8.28	2.46 ± 0.48	10.7	ND	ND	0.24 ± 0.02	2.36
**C17:1**	1.12 ± 0.81	5.75	0.60 ± 0.35	2.74	1.10 ± 1.20	5.19	ND	ND	ND	ND	ND	ND	ND	ND	ND	ND	1.23 ± 0.00	4.28	ND	ND	ND	ND	ND	ND
**C18:1n9**	3.68 ± 2.37	18.8	3.78 ± 1.03	17.3	2.67 ± 3.04	12.6	1.86 ± 0.58	17.8	2.88 ± 1.97	16.3	2.85 ± 1.87	16.8	5.81 ± 2.40	21.4	4.21 ± 1.15	20.1	4.47 ± 3.48	15.6	5.01 ± 0.73	21.8	0.63 ± 0.15	10.4	1.35 ± 0.31	13.4
**C20:1**	0.56 ± 0.13	2.84	0.67 ± 0.22	3.04	0.70 ± 0.27	3.26	ND	ND	0.60 ± 0.16	3.40	0.64 ± 0.00	3.76	0.71 ± 0.00	2.61	0.70 ± 0.04	3.32	ND	ND	0.62 ± 0.16	2..69	0.24 ± 0.02	3.98	ND	ND
**C22:1n9**	ND	ND	0.78 ± 0.21	3.56	0.60 ± 0.00	2.81	0.28 ± 0.00	2.71	ND	ND	ND	ND	ND	ND	ND	ND	ND	ND	ND	ND	ND	ND	0.28 ± 0.00	2.76
**C24:1**	ND	ND	ND	ND	0.74 ± 0.00	3.49	ND	ND	ND	ND	ND	ND	ND	ND	ND	ND	ND	ND	ND	ND	ND	ND	ND	ND
**ΣMUFA**	7.64 ± 1.99	39.1	7.49 ± 1.50	34.2	7.83 ± 2.05	36.8	3.11 ± 0.92	29.9	4.99 ± 1.74	28.3	4.70 ± 1.63	27.7	8.44 ± 2.71	31.1	6.78 ± 1.61	32.3	8.08 ± 2.85	28.1	8.09 ± 1.86	35.1	0.87 ± 0.22	14.4	1.86 ± 0.61	18.6
**C18:2n6t**	ND	ND	0.31 ± 0.00	1.39	ND	ND	ND	ND	ND	ND	0.25 ± 0.00	1.49	ND	ND	ND	ND	0.61 ± 0.00	2.12	ND	ND	ND	ND	ND	ND
**C18:2n6c**	0.36 ± 0.12	1.82	ND	ND	0.41 ± 0.00	1.90	0.28 ± 0.02	2.69	0.43 ± 0.20	2.43	0.37 ± 0.12	2.19	0.57 ± 0.00	2.08	0.80 ± 0.09	3.80	0.71 ± 0.00	2.45	0.51 ± 0.00	2.23	ND	ND	0.25 ± 0.02	2.49
**C18:3n6**	0.23 ± 0.00	1.18	0.49 ± 0.00	2.23	ND	ND	ND	ND	0.50 ± 0.00	2.82	0.32 ± 0.09	1.89	ND	ND	ND	ND	ND	ND	ND	ND	0.30 ± 0.06+	5.01	0.28 ± 0.02	2.81
**C20:3n6**	ND	ND	ND	ND	ND	ND	ND	ND	ND	ND	ND	ND	ND	ND	ND	ND	ND	ND	ND	ND	0.27 ± 0.00	4.48	ND	ND
**C20:4n6**	ND	ND	ND	ND	ND	ND	ND	ND	ND	ND	ND	ND	ND	ND	ND	ND	ND	ND	ND	ND	ND	ND	1.37 ± 0.00	13.6
**ΣPUFA n6**	0.59 ± 0.12	3.00	0.79 ± 0.13	3.62	0.41 ± 0.00	1.90	0.28 ± 0.02	2.69	0.93 ± 0.15	5.25	0.95 ± 0.10	5.57	0.57 ± 0.00	2.08	0.80 ± 0.09	3.80	1.32 ± 0.07	4.57	0.5 ± 0.00	2.23	0.58 ± 0.04	9.49	1.90 ± 0.33	18.9
**C18:3n3**	ND	ND	1.54 ± 0.00	7.03	ND	ND	ND	ND	ND	ND	ND	ND	ND	ND	ND	ND	ND	ND	ND	ND	0.56 ± 0.07	9.15	0.51 ± 0.00	5.04
**C20:3n3**	ND	ND	0.85 ± 0.00	3.89	ND	ND	ND	ND	ND	ND	ND	ND	ND	ND	ND	ND	ND	ND	ND	ND	ND	ND	0.34 ± 0.04	3.34
**C20:5n3**	0.60 ± 0.16	3.08	0.72 ± 0.30	3.31	0.50 ± 0.14	2.37	0.65 ± 0.30	6.25	0.68 ± 0.26	3.85	0.81 ± 0.47	4.76	1.06 ± 0.51	3.89	1.37 ± 0.39	6.50	1.90 ± 1.14	6.61	0.77 ± 0.00	3.34	0.37 ± 0.11	6.10	0.82 ± 0.33	8.14
**C22:6n3**	ND	ND	0.70 ± 0.12	3.19	ND	ND	0.53 ± 0.21	5.07	0.54 ± 0.13	3.05	0.68 ± 0.31	4.00	0.90 ± 0.40	3.30	1.36 ± 0.39	6.49	1.64 ± 1.26	5.69	0.86 ± 0.00	3.75	0.35 ± 0.11	5.81	0.72 ± 0.24	7.21
**ΣPUFA n3**	0.60 ± 0.16	3.08	3.82 ± 0.33	17.4	0.50 ± 0.14	2.37	1.18 ± 0.26	11.3	1.22 ± 0.21	6.90	1.49 ± 0.39	8.75	1.06 ± 0.45	7.19	2.73 ± 0.48	12.9	3.53 ± 0.07	12.3	1.63 ± 0.07	7.09	1.28 ± 0.13	21.1	2.38 ± 0.31	23.7
**Σ PUFA**	1.19 ± 0.19	6.07	4.61 ± 0.34	21.0	0.91 ± 0.12	4.27	1.46 ± 0.26	14.0	2.14 ± 0.21	12.2	2.43 ± 0.37	14.3	1.62 ± 0.45	9.27	3.53 ± 0.49	16.8	4.85 ± 1.17	16.9	2.14 ± 0.18	9.32	1.86 ± 0.11	30.6	4.28 ± 0.33	42.6
**Σ Fatty Acid**	19.6 ± 2.83	100	21.90 ± 1.89	100	21.30 ± 3.21	100	10.43 ± 1.34	100	17.6 ± 2.55	100	16.9 ± 1.95	100	27.2 ± 3.61	100	21.0 ± 2.14	100	28.8 ± 3.84	100	22.9 ± 3.16	100	6.08 ± 0.45	100	10.0 ± 0.41	100

**Notes.**

$\bar {X}$, Mean; SD, Standard Deviation; %, Percentage; SFA, Saturated Fatty Acid; MUFA, Monounsaturated Fatty Acid; PUFA, Polyunsaturated Fatty Acid; ND, Not Detected.

Regarding LB individuals, males in both locations in the Chilean SEPO (Coquimbo, Concepción; 30°S, 36°S) presented the same record of FAs (15 types). However, the location with the highest content of total FAs was Coquimbo with 7.84 ± 0.52 mg*g DW^−1^ *vs.* Concepción with 5.22 ± 0.56 mg*g DW^−1^. In turn, in LB non-ovigerous females, FA analyses were not performed due to the absence of these specimens in these areas (Coquimbo, Concepción) during the capture period. Regarding LB ovigerous females, the greatest variety of FAs was recorded in Concepción, with 17 types of FAs, corresponding to the content of 10.04 ± 0.41 mg*g DW^−1^.

Along the latitudinal gradient, SP individuals captured in the localities of Marcona (15°S), Chala (16°S), Planchada (16°34′S), and Mollendo (17°S) presented the highest saturated fatty acid (SFA) contents. In turn, the monounsaturated fatty acids (MUFAs) decreased from furthest latitudes (09°S and 36°S) towards latitudes centrals in Perú such as Lima and Lomitas in the SEPO. Regarding the PUFA-n6, males presented trace amounts in the localities of Chimbote (09°S), Huacho (11°S), Cañete (13°S), and Lomitas (14°S), while females (ovigerous and non-ovigerous) showed a high content in the localities of Planchada (16° 34′S) and Mollendo (17°S). A notable increase in the PUFA-n3 content was observed from 09°S LAT towards 17°S LAT in the Peruvian SEPO, with a greater abundance in the Planchada locality. While the Mollendo locality presented a low content of PUFA-n3 in females (ovigerous and non-ovigerous), and an absence of these FAs in the Huacho (11°S) (males and non-ovigerous females) and Chimbote localities (09°S) (males) (Fig. S1, Tables 3–5).

In turn, the SFA, MUFA and PUFA contents in all of the evaluated *G. monodon* individuals along the latitudinal gradient of the Chilean SEPO presented significant differences between the localities of Coquimbo (30°S) and Concepción (36°S), and also between sexes (male, ovigerous female) (Tables 3–5). Here, the SFA content presented similar values between these two localities and sexes, while the MUFA content was higher in the Concepción locality for both males and ovigerous females. The PUFA-n6 and PUFA n-3 contents were consistently higher in ovigerous females than males for both of these localities (Fig. S1, Tables 3–5).

**Figure 6:**
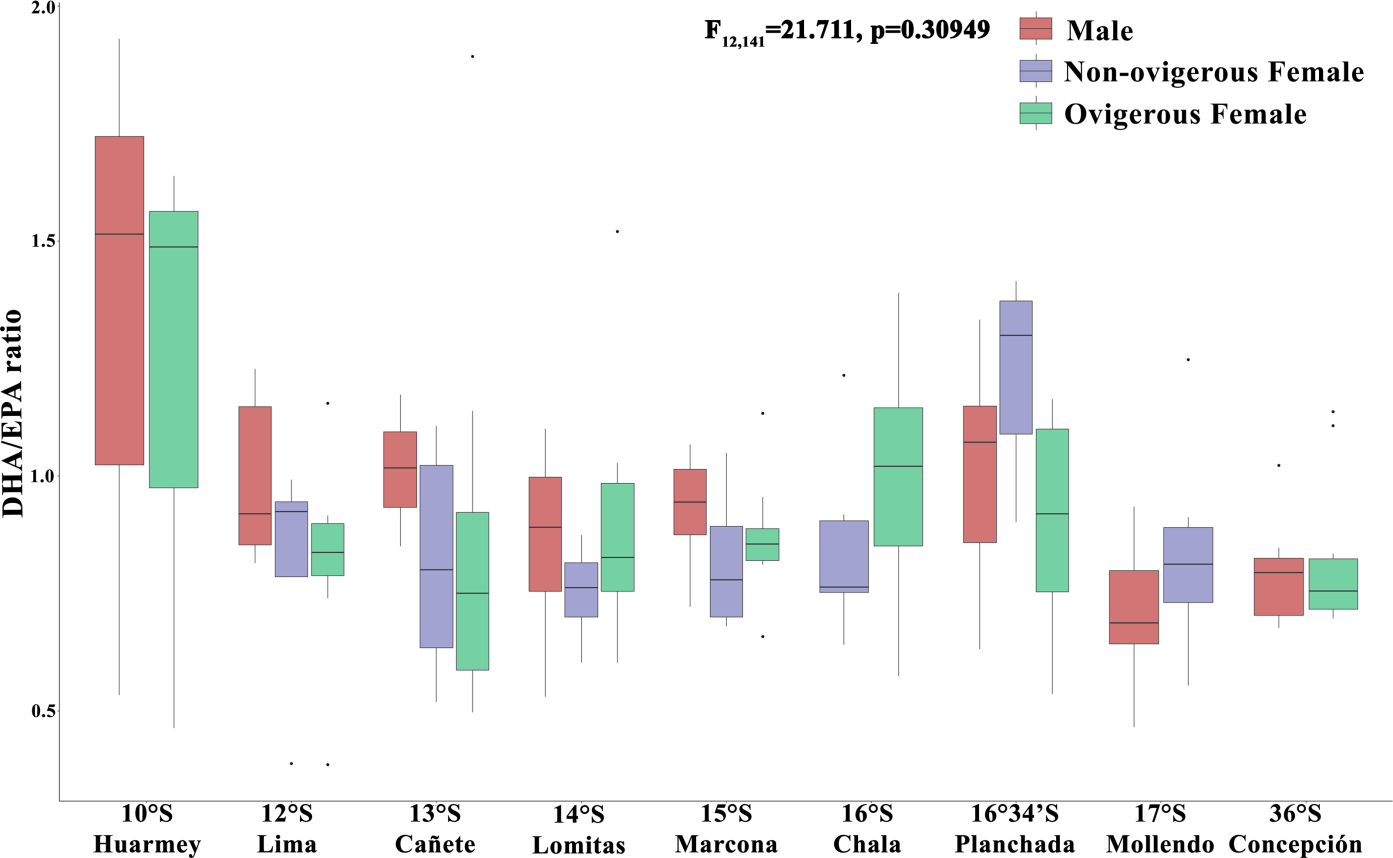
Nutritional condition index (DHA/EPA ratio) variability of *Grimothea monodon* in their lifestyles: “small-pelagic (SP)” (09°S–17°S) and “large-benthic (LB)” (30°S–36°S) in the Southeast Pacific Ocean.

In turn, the DHA/EPA ratio, considered as an index of the nutritional condition (Fig. 6), showed values between 0.36–3.54 along the latitudinal gradient. In particular, the absence of the DHA/EPA ratio in some localities (Chimbote, Huacho, Coquimbo) is due to the presence of only one type of essential fatty acid (DHA or EPA) recorded in the samples analyzed. For this ratio, the highest values were found in all individuals from the Huarmey locality (10°S) (range: 0.46–1.92) and the non-ovigerous females from the Planchada locality (16° 34′S) (1.21 ± 0.22), while the lowest values were found in males from the Mollendo locality (17°S) (0.71 ± 0.12) and ovigerous females from Concepcion (36°S) (0.71 ± 0.12). The DHA/EPA ratio along the latitudinal gradient presented no significant differences for the interaction between the factors locality*sex (*F*_12,141_ = 1.17, *p* = 0.309) or the factor of sex alone (*F*_2,141_ = 1.171, *p* = 0.5767); the only significant differences were recorded for the locality factor (*F*_8,141_ = 2.86, *p* = 0.006).

**Figure 7:**
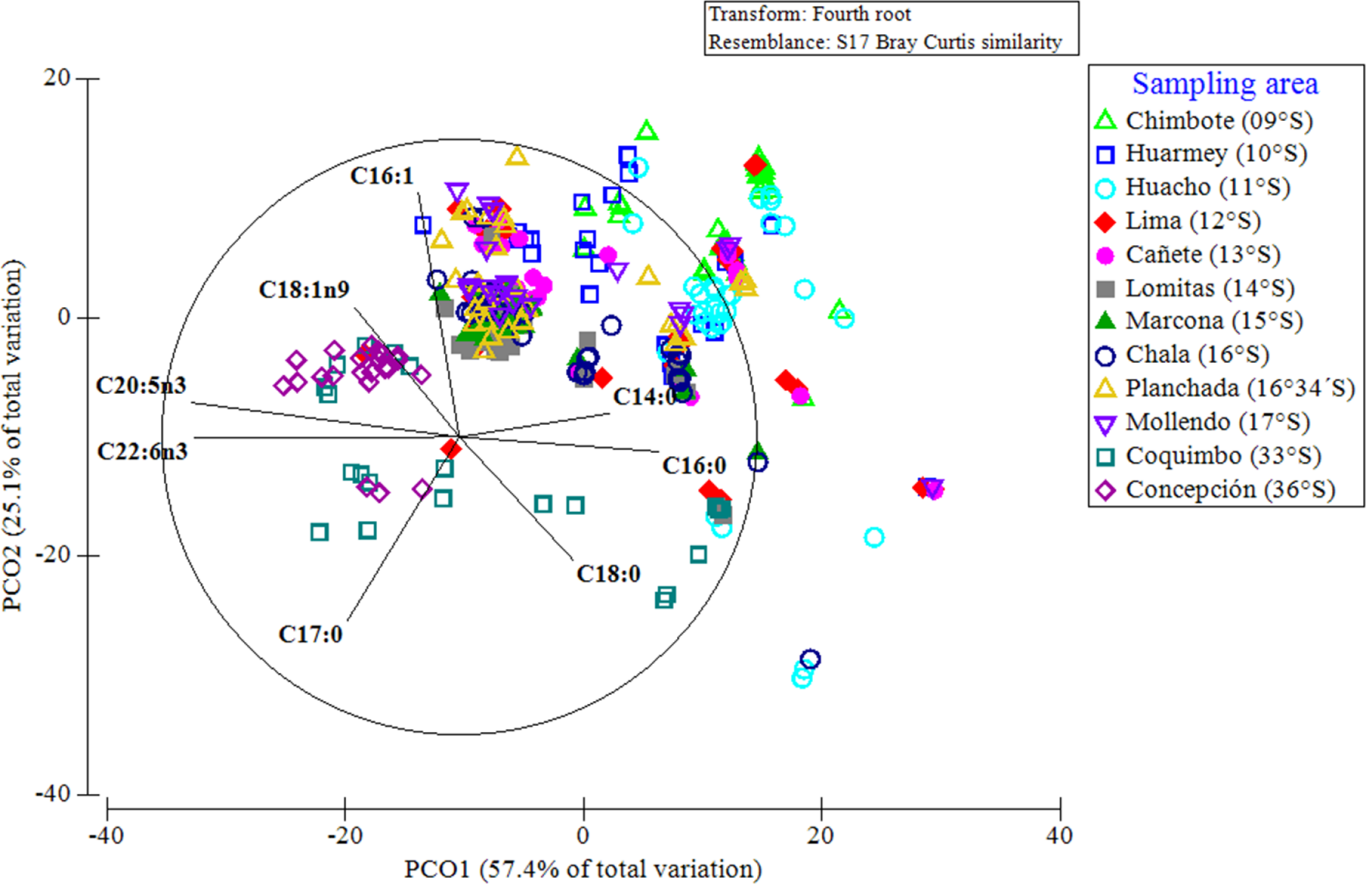
Principal coordinate analysis (PCoA) of the fatty acid profile of *Grimothea monodon* in their lifestyles: “small-pelagic (SP)” (09°S–17°S) and “large-benthic (LB)” (30°S–36°S).

A PCoA considering the entire data set of FAs, indicated that 83% of the differences in the FA profiles of all of the evaluated red squat lobsters were explained by the latitudinal variations (Fig. 7). In this multivariate ordination analysis, a clear spatial separation between the SP (from 9°S to 17°S) and LB (from 33°S to 36°S) groups was observed. In the SP individuals, a group differentiation between the FA profiles of the different Peruvian localities was also observed, mainly between the northern (Chimbote, Huarmey, Huacho) and southern (Marcona, Chala, Planchada, Mollendo) localities. The FAs that most contributed to these differences and/or groupings in the FA profiles of SP individuals according to localities (by SIMPER), were the SFAs (C14:0, C16:0) with 13%–25% and the MUFAs (C16:1, C18:1n9) between 10%–20%. In turn, for the LB individuals of the Chilean localities, the SFAs C18:0 (12%–16%) together with PUFAs C20:5n3 (15%) and C22:6n3 (12%) were the FAs that most contributed to this differentiation in the FA profiles (Table S3). Finally, the differences in the FA profiles of the different localities, verified by a 2-way PERMANOVA test, revealed a significant effect of the interaction between locality*sex (Pseudo-*F*_11,345_ = 3.615, *p* = 0.001). This finding was verified by the ANOSIM test, with an overall value of *R* = 0.422 and a significance level of 0.001 (Table S4).

## Discussion

Within the global studies of latitudinal variations in biological traits of marine ectotherms with extensive distributions along a climatic gradient, our study reveals the changes in the integrated bioenergetic condition (CL, W, Kn, biochemical constituents) of the red squat lobster (*G. monodon*) throughout its wide latitudinal distribution in the SEPO. In this context, the CL variability of this species along the latitudinal gradient can be linked to the physicochemical parameters of the environment (temperature, salinity, oxygen), which vary latitudinally in the SEPO (Roa & Tapia, 1998; Mazumder et al., 2016). The combined effect of these parameters on marine organisms can be considered as selective pressures of the environment (Assan et al., 2020; Hsieh, Tew & Meng, 2023). Thus, as observed in our study, variations in environmental conditions could be determining the relationship between the size of individuals, their sexual maturity, and reproductive status along a latitudinal gradient (Assan et al., 2020).

Small pelagic (SP) (Chimbote-Mollendo) ovigerous females were consistently observed to have sizes over 20 mm CL at latitudes of 09–12°S and 11 mm CL at latitudes of 17°S. This trend is similar to that reported by Franco-Meléndez (2012), who considered small individuals (∼11 mm CL) as juveniles with early maturity. Large benthic (LB) ovigerous females were recorded with sizes over 45 mm CL in Coquimbo (30°S) and 63 mm CL in Concepción (36°S), values were different to those reported in previous studies for this species on the coast of Chile (Guzmán-Rivas et al., 2021b). Considering that in crustacean fishery management models, the states of first sexual maturity and reproductive categories (mature and immature) are related to size (CL), environmental seasonality (Hernáez & Wehrtmann, 2011; Peixoto et al., 2018), and fishing impact (Peixoto et al., 2018), future studies must complement our findings with in-depth analyses of gonadal development during an annual cycle as reported by Flores et al. (2020).

Continuing in a reproductive and sexual context, our findings show an increase in sex ratio values in SP individuals, consistent with previous reports by Franco-Meléndez (2012). This increase may be attributed to minimal or low fishing pressure on SP populations compared to LB populations, where our results indicate that sex ratio values for females are lower than for males. This pattern is presumably influenced by the constant industrial fishing pressure on LB populations along the coast of Chile (SUBPESCA, 2023). However, these observations regarding LB sex ratios should be interpreted with caution due to the low sample size analyzed. A recent report on biological and fisheries monitoring of LB populations of *G. monodon* conducted by IFOP (2025) indicates a generally higher proportion of females relative to males, except in the Coquimbo area, where males are more abundant than females.

To estimate the biomass and growth of resources, the length-weight relationship of organisms is preferably considered as the function with the greatest contribution in biological-fishery models (Cifuentes et al., 2012; Soinski et al., 2020). However, this type of relationship alone is insufficient, so it is necessary to reinforce it through other parameters such as the relative condition (Kn) and the type of growth (isometric *vs.* allometric) found in natural populations (Froese, 2006; Noori et al., 2015; Khademzadeh & Haghi, 2017; Paramo, Rodriguez & Quintana, 2024). Our results established the isometric or allometric growth presented by red squat lobster populations from different locations. Based on the equation (W = aL^b^) described by Paramo, Rodriguez & Quintana (2024), a population with isometric growth (or “equal growth rates of body sections”)) is composed of individuals that present “b” values between 2.5 and 3.5, while populations with positive or negative allometric growth have individuals with “b” values of >3.5 and <2.5, respectively. In this sense, in our results, all of the evaluated individuals from the localities of Chimbote (09°S), Huarmey (10°S), Huacho (11°S) and Mollendo (17°S) indicating that individuals from these populations or localities increased more in weight (W) than size (CL). On the contrary, individuals from the populations of Chala (16°S) and Concepción (36°S) showed that those individuals presented a greater increase in their CL than their W (for details see Jisr et al. (2018) and Valencia-Cayetano et al. (2023). It should be noted that the allometry observed in individuals of *G. monodon* from the localities of Chala and Concepción may be a fitness response to local environmental conditions, as well as a result of the strong fishing pressure this resource is exposed to in both areas (De Carvalho-Souza et al., 2023).

In turn, the relative condition factor helps us identify the health status of natural populations, and interpret the body condition of adult individuals in highly variable environments (Fitzgerald et al., 2002; Froese, 2006; Araujo et al., 2021; De Carvalho-Souza et al., 2023). In our findings, and in agreement with the lifestyles of *G. monodon* adults in the SEPO (small and pelagic: SP; large and benthic: LB), all of the evaluated individuals presented a “good condition factor”. In agreement with the above, similar values and categorizations of Kn have also been reported in other crustaceans of the Munididae family from temperate latitudes (Paramo, Rodriguez & Quintana, 2024). In turn, when comparing between lifestyles of adult individuals (SP *vs.* LP), slight variations in Kn were found. These differences could be plausibly explained by the combined effect of environmental factors (mainly temperature and nutrient availability) on key physiological processes (growth and reproductive maturation) that determine the body condition of ectothermic invertebrates such as *G. monodon* (Roa & Tapia, 1998; Bascur et al., 2017; Guzmán-Rivas, Quispe & Urzúa, 2022). In this energetic and reproductive context, *G. monodon* has demonstrated an adaptive biochemical strategy that includes storing large amounts of energy reserves to sustain several egg laying events during an annual cycle under variable environmental conditions and food availability (Montecino & Lange, 2009; Flores et al., 2020; De Carvalho-Souza et al., 2023). This strategy could strongly influence the nutritional condition of *G. monodon* due to changes in the availability and type of planktonic food, as indicated by chlorophyll-a concentrations (Chl-a). These variations are driven by the continuity and intensity of coastal upwellings along the Humboldt Current (Hutchings et al., 1995; Franz et al., 2012; Deville et al., 2020; Pérez-Aragón et al., 2025) and are reflected in decapods by the amount of energy reserves stored in their bodies (Rosa & Nunes, 2003). Additionally, differences in feeding preferences between the two morphotypes (SP *vs.* LB) should be considered, with pelagic individuals primarily consuming plankton and benthic individuals feeding on detritus (Kiko et al., 2015; Guzmán-Rivas et al., 2021b; Yapur-Pancorvo et al., 2023).

The bioenergetic condition, based on proximal biochemical composition, is considered an integrative physiological indicator that reflects the nutritional status of marine invertebrates (Guzmán-Rivas et al., 2021a). Nonetheless, to date this indicator has scarcely been included in the exploitation models (age-size structured assessment) and management plans of crustacean fisheries (Castillo-Vargasmachuca et al., 2017). Our results showed significant variability in the biochemical composition (glucose, proteins, lipids, FAs) of *G. monodon* throughout its latitudinal distribution in the SEPO, which in turn significantly relates to the dual body traits of adult individuals (SP, LB). As revealed in this study, the varying contents of the biochemical constituents found in *G. monodon* can most likely be linked to the development of physiological and biochemical strategies necessary to face the environmental fluctuations that occur throughout the diverse conditions along the latitudinal gradient (Vernberg, 1962; Garvey et al., 2003; Gaitán-Espitia et al., 2017). In this context, *G. monodon* is most likely generating strategies focused on the optimal use and storage of nutrients or essential biomolecules, whose abundance in the natural environment vary along the latitudinal gradient of the SEPO. The amount of nutrients or essential biomolecules is influenced by the combined effects of physicochemical parameters (temperature, salinity, oxygen), which delimit the minimum oxygen zones, as well as the intensity of upwellings that occur throughout the SEPO (Hutchings et al., 1995; Franz et al., 2012).

In decapods, glucose is considered a bioenergetic molecule of immediate use, and variations in its levels and/or content are considered an indicator of well-being or stress in individuals (Viña-Trillos, Guzmán-Rivas & Urzúa, 2021). This molecule helps regulate the consumption of other bioenergetic fuels (such as lipids and proteins), which are used to face adverse environmental conditions (Mozsár et al., 2015). Our findings indicated that glucose levels in SP individuals could be influenced by multiple environmental stressors found in this area of the Peruvian SEPO, including: (i) a warm water temperature (Flores et al., 2013), (ii) a continuous upwelling, but with low productivity (Montecino & Lange, 2009; Deville et al., 2020), and (iii) a greater predominance and extension of the minimum oxygen zone (Fuenzalida et al., 2009). On the other hand, the glucose levels of LB individuals could indicate that they are exposed to suboptimal environmental conditions in the Chilean SEPO, characterized by: i) cold temperatures, ii) a higher amount of dissolved oxygen, and iii) a strong seasonality in the availability of nutrients (Deville et al., 2020). Another plausible explanation for the variability of this important carbohydrate is its high demand as an energy substrate in the physiological process of molting (Wang, Li & Chen, 2016; Li et al., 2021), which presents a higher frequency and rate in decapod individuals that are exposed to high and/or warm temperatures, as has been reported in *G. monodon* individuals from northern Perú near the equatorial zone (Liu et al., 2022; Khalsa et al., 2023).

Proteins are important molecules involved in the muscle growth and regeneration of decapods (Zuloaga et al., 2018). Their content is closely linked to the availability of nutrients present in the SEPO upwelling systems; this availability varies greatly along the latitudinal gradient (Fraser & Rogers, 2007; Bascur et al., 2017; Guzmán-Rivas et al., 2021a; Zuloaga et al., 2023). In our findings, a higher protein content in SP individuals in northern Perú (9°S–14°S) compared to those in southern Perú (15°S–17°S) and LB individuals in Chile (30°S–36°S), can be explained by the fact that in northern Perú, warm temperatures and a high availability of planktonic food predominate due to a continuous and nutrient-rich upwelling (Montecino & Lange, 2009; Deville et al., 2021). These oceanographic conditions allow SP individuals to maintain locomotor activity (swimming in the water column) (Kiko et al., 2015), contrary to LB individuals that spend some time immobile in the benthic zone (Yapur-Pancorvo et al., 2023). Coincidentally, LB individuals have a protein content similar to SP individuals from southern Perú. This convergent biochemical response could be related to the combined effects of cold temperatures and strong temporal variations in food availability (product of seasonal upwelling) in these areas of the SEPO (Deville et al., 2021), which could promote slow growth in LB individuals characterized by only one molting season in spring/summer during an annual cycle (Palma & Arana, 1997; SUBPESCA, 2008).

At the sex level, in most of the study locations in the SEPO, females (ovigerous and non-ovigerous) had higher protein and lipid contents than males. This trend was similar to that reported by Guzmán-Rivas et al. (2021a). These authors indicate that inter-sexual variations in the amount of these biochemical constituents could be particularly beneficial for females, which have rapid growth and a very costly reproductive process in terms of energy. Contrary to males, females must invest considerable energy reserves (mainly lipids) in the production of numerous eggs, which are incubated under their abdomens for long periods throughout embryogenesis until larval hatching occurs (Guzmán-Rivas et al., 2021a).

Lipids, as the main source of energy reserve in decapods (Bascur et al., 2017; Viña-Trillos, Guzmán-Rivas & Urzúa, 2021), are considered an important indicator of the bioenergetic and/or nutritional status of individuals. Our study reveals how this energy reserve can modulate both the lifestyles of *G. monodon* (SP, LB) and the nutritional condition of its natural populations throughout the SEPO (Cleary, Bradford & Janz, 2012; Mozsár et al., 2015). Our results show that changes in the lipid content are related to notable variations in environmental conditions along the latitudinal gradient, with a particularly abrupt change in sea temperatures that is linked to upwelling zones at 12°S (Lima) (Igarza et al., 2019; Deville et al., 2021; Gonzalez-Pestana, Alfaro-Shigueto & Mangel, 2022). In our study, it was also evident that the amount of lipids varied in both SP and LB individuals along the gradient. This can be postulated as a presumably adaptive biochemical response to sustain the energy expenditure of individuals based on their lifestyle (SP, pelagic: greater swimming activity *vs.* LB, benthic: less swimming activity), and also to face the seasonal availability of nutrients in the localities of Coquimbo and Concepción (Deville et al., 2021); which together, through analyses of the FA profiles, also reflects the type of food or prey items of these individuals (SP: microalgae; LB: sedimentary material) (Franco-Meléndez, 2012; Guzmán-Rivas et al., 2021b; Yapur-Pancorvo et al., 2023).

In decapod crustaceans, the FA profile depends on the type of diet or prey consumed; these carbon and hydrogen molecules are conservatively deposited in the different types of tissues and/or organs (gonad, hepatopancreas or muscle) (Viña-Trillos, Guzmán-Rivas & Urzúa, 2021; Feng et al., 2023), and their content can vary at the intra-individual level depending on the organ where they are stored, and also due to physiological processes such as molting, regeneration of body structures or reproduction (Dvoretsky et al., 2024). In our findings, similar to the trends recorded in the biomolecules mentioned above (glucose, proteins and lipids), the total FAs found in the muscle tissue of *G. monodon* individuals showed highly contrasting contents and compositions in: “small pelagic, SP” *vs.* “large benthic, LB” individuals (see Tables 3–5). As mentioned above, variations in the FA profile could be linked to the type of available food that individuals consume according to their lifestyle (Sahu et al., 2013; Cañavate, 2019; Resmi et al., 2023), which is explained through the contribution of FA biomarkers such as SFAs (C14:0, C16:0) in SP individuals and PUFAs (C20:5n3, C22:6n3) in LB individuals. Furthermore, considering the importance of establishing how these molecules are distributed and stored in the organs as a bioenergetic source to carry out various key physiological processes (growth, reproduction, mobility, survival) (Yuan et al., 2019; Dvoretsky et al., 2024), future comparative studies should include other storage organs (hepatopancreas) that could aid in elucidating the functionality of these molecules in the growth and reproduction processes of this species (Bascur et al., 2017; Guzmán-Rivas et al., 2021b; Barriga-Sánchez et al., 2023).

In a context of functional ecology of FAs, our findings revealed a higher presence of structural-type SFAs (mainly C16:0) in SP individuals from low-latitude locations with warm temperatures (Chimbote, Huacho), and a greater predominance of PUFAs (mainly C20:5n3 (EPA) and C22:6n3 (DHA)) in LB individuals from high-latitude locations (Concepción) (Guzmán-Rivas et al., 2021b). A greater predominance of SFAs could allow *G. monodon* to maintain homeostasis and stable cell membranes under warm temperatures at low latitudes, while the increase in PUFAs towards high latitudes with cold temperatures and reduced seawater salinities could allow *G. monodon* to maintain the fluidity of its cell membranes (avoid freezing) through a complex physiological process of homeoviscous adaptation (For studies in decapods, see: Chapelle (1978) and Romano et al. (2014). Also, maintaining the fluidity of its cell membranes allows *G. monodon* to develop strategies for the accumulation of energy reserves under adverse environmental conditions characterized by cold temperatures and prolonged periods of absence of planktonic food, as occurs at high latitudes in the SEPO (Guzmán-Rivas et al., 2021b).

In general, decapod crustaceans do not have a high capacity to biosynthesize essential omega-3 PUFAs (EPA, DHA) and, therefore, these complex biomolecules must be obtained exclusively from their diet, to subsequently be used for growth and reproduction (Jónasdóttir, 2019). Furthermore, the availability of these essential FAs in the environment depends largely on key environmental factors (temperature and oxygen), as well as oceanographic phenomena (upwellings) (Linder et al., 2010; Jónasdóttir, 2019). In this sense, considering an ecosystemic approach, the content of these essential FAs in *G. monodon*, and particularly their ratio values (DHA/EPA) can reflect the nutritional status of individuals in real time along their latitudinal distribution gradient in the SEPO. In our results, the DHA/EPA index consistently presented values close to or greater than 1, indicating that *G. monodon* populations along the SEPO latitudinal gradient develop within a marine system shaped by environmental variability and anthropogenic impacts (Pérez-Aragón et al., 2025). Similar DHA/EPA values have been reported by Guzmán-Rivas et al. (2021b) for *G. monodon* from the SEPO (DHA/EPA: 1.03–0.99; 29°S–36°S), and also for *Portunus trituberculatus* (0.70 and 0.84) from Indo-West Pacific (Hu et al., 2017).

## Conclusions

Our findings demonstrate clear differences in the integrated bioenergetic condition of the two morphotypes of the red squat lobster (*G. monodon*) across its broad latitudinal distribution in the SEPO (Chimbote–Concepción). Environmental variability along this gradient, driven by factors such as temperature, dissolved oxygen, upwelling, and food availability likely promotes contrasting phenotypic responses (pelagic *vs.* benthic morphotypes) and shapes distinct strategies for storing and utilizing energy reserves to maintain nutritional condition.

The condition factor proved useful as a low-cost, noninvasive tool for monitoring the natural state of *G. monodon* populations and should be incorporated into fisheries assessments. However, our results also show that relying solely on morphometric parameters (size and weight) in size–age structured fishery models is insufficient. Comprehensive monitoring of nutritional condition requires integrating biochemical indicators (lipids, proteins, carbohydrates, fatty acids) with intra-individual traits (sex, reproductive status, Kn index).

We recommend that management and exploitation models for *G. monodon* and other bioresources in the SEPO adopt a holistic approach that combines physiological, nutritional, and ecological perspectives. Incorporating these indicators into fishery models will allow real-time evaluation of population health and support the sustainable exploitation of this resource throughout the SEPO.

## Supplemental Information

10.7717/peerj.20339/supp-1 Supplemental Information 1 Fatty acid (expressed in mg FA g DW^−1^) in males, non-ovigerous females, and ovigerous females of *Grimothea monodon* individuals: “small- pelagic (SP)” (09°S–17°S) and “large-benthic (LB)” (30°S–36°S)

10.7717/peerj.20339/supp-2 Supplemental Information 2 Sampling locations of *Grimothea monodon* individuals: “small-pelagic (SP)” (09°S–17°S) and “large-benthic (LB)” (30°S–36°S) present in the Southeastern Pacific Ocean

10.7717/peerj.20339/supp-3 Supplemental Information 3 Environmental parameters of the Southeastern Pacific Ocean: sea surface temperature, dissolved oxygen, salinity, and chlorophyll obtained from IMARPE, IFOP, and the web platforms Giovanni and Copernicus

10.7717/peerj.20339/supp-4 Supplemental Information 4 Analysis of the percentage of similarity (SIMPER) of *Grimothea monodon* individuals: “small- pelagic (SP)” (09°S–17°S) and “large-benthic (LB)” (30°S–36°S) captured in the Southeastern Pacific Ocean Note: Av.Abund, Average abundance; Av.Sim, Average similarity; Contrib %, Contribution Percentage; Cum %, Cummulative Contribution Percentage

10.7717/peerj.20339/supp-5 Supplemental Information 5 Analysis of similarity (ANOSIM) of *Grimothea monodon* individuals: “small- pelagic (SP)” (09°S–17°S) and “large-benthic (LB)” (30°S–36°S) captured in the Southeastern Pacific Ocean

10.7717/peerj.20339/supp-6 Supplemental Information 6 Raw data: all environmental parameters along latitudinal gradient

10.7717/peerj.20339/supp-7 Supplemental Information 7 Raw data: morphometric traits, relative condition and biochemical condition This data was used for statistical analysis to compare between the different localities and morphotypes along latitudinal gradient in the Southeast Pacific Ocean.

## Additional Information and Declarations

### Data Availability

The following information was supplied regarding data availability: The raw measurements are available in the Supplementary Files.
