# Supplementary figures and images for: Latitudinal variations in morphometric traits and bioenergetic status of adult red squat lobsters Grimothea monodon (H. Milne Edwards, 1837) in the Southeast Pacific Ocean
Source: PeerJ. 2025 Nov 17;13:e20339. doi: 10.7717/peerj.20339 (PMC12633147; doi:10.7717/peerj.20339)

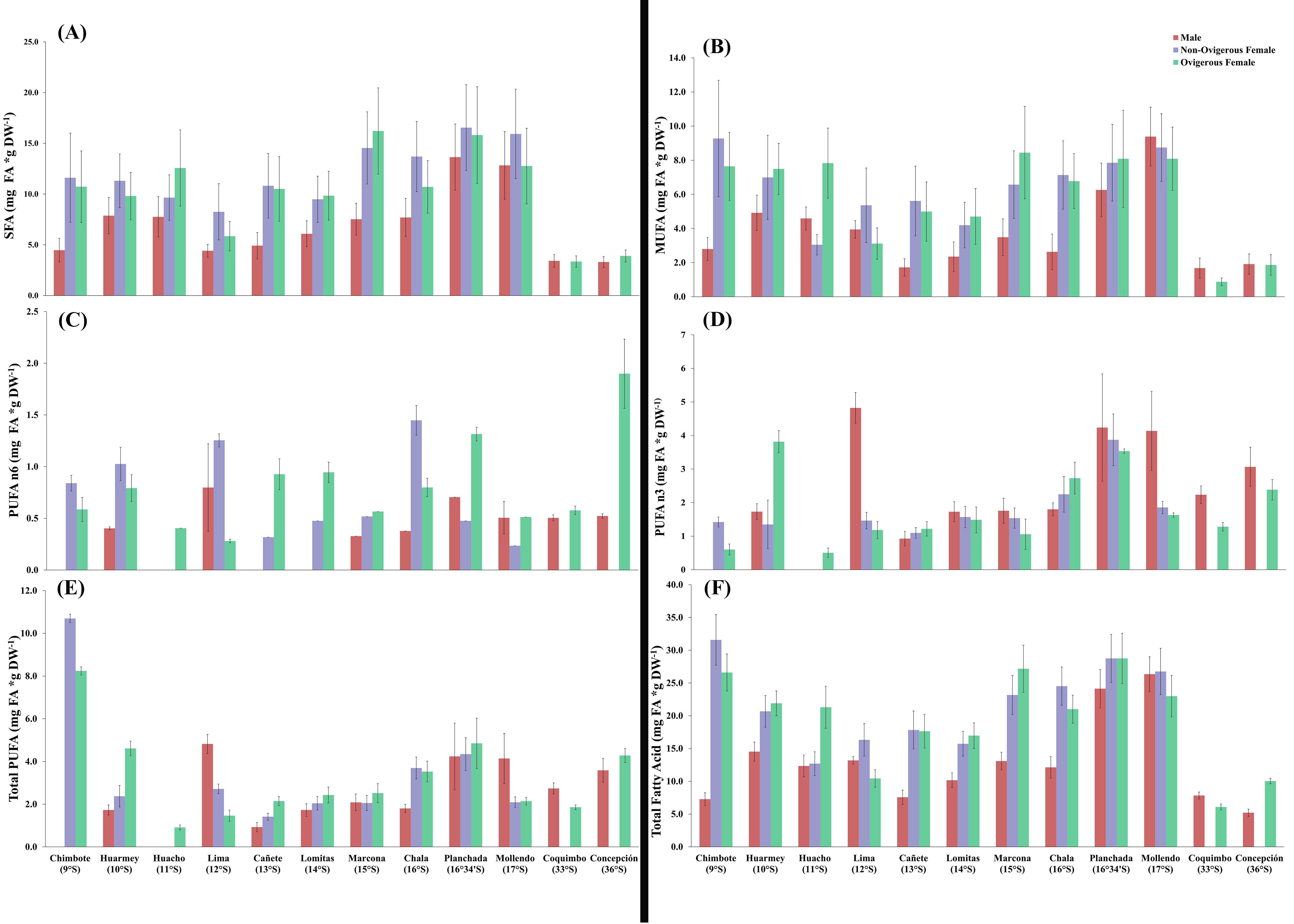

Supplement: Supplemental Information 1 [file peerj-13-20339-s001.png]
