# Supplementary material for: Latitudinal variations in morphometric traits and bioenergetic status of adult red squat lobsters Grimothea monodon (H. Milne Edwards, 1837) in the Southeast Pacific Ocean
Source: PeerJ. 2025 Nov 17;13:e20339. doi: 10.7717/peerj.20339 (PMC12633147; doi:10.7717/peerj.20339)
Supplement: Supplemental Information 2 [file peerj-13-20339-s002.docx]

**Table S1:** Sampling locations of *Grimothea monodon* individuals: “small-pelagic (SP)” (09°S-17°S) and “large-benthic (LB)” (30°S-36°S) present in the Southeastern Pacific Ocean

| **Small**  **Pelagic** | **Chimbote** | 09° 29.1' 0.00'' S 78° 28.6' 0.00'' W |
| --- | --- | --- |
|  | **Huarmey** | 10° 08.7' 0.00'' S 78° 36.0' 0.00'' W |
|  | **Huacho** | 11° 19.4' 0.00'' S 78° 17.6' 0.00'' W |
|  | **Lima** | 12° 27.8' 0.00'' S 77° 13.5' 0.00'' W |
|  | **Cañete** | 13° 10.8' 0.00'' S 76° 47.5' 0.00'' W |
|  | **Lomitas** | 14° 46.5' 0.00'' S 75° 49.9' 0.00'' W |
|  | **Marcona** | 15° 16.3' 0.00'' S 75° 20.2' 0.00'' W |
|  | **Chala** | 16° 24.9' 0.00'' S 74° 18.4' 0.00'' W |
|  | **Planchada** | 16° 34.0' 0.00'' S 73° 25.0' 0.00'' W |
|  | **Mollendo** | 17° 8.80' 0.00'' S 72° 1.10' 0.00'' W |
| **Large Benthic** | **Coquimbo** | 30° 12' 52.16'' S 72° 37' 33.20'' W |
|  | **Concepción** | 36° 52.0' 50.0'' S 73° 38.0' 55.0'' W |
