# Supplementary material for: Latitudinal variations in morphometric traits and bioenergetic status of adult red squat lobsters Grimothea monodon (H. Milne Edwards, 1837) in the Southeast Pacific Ocean
Source: PeerJ. 2025 Nov 17;13:e20339. doi: 10.7717/peerj.20339 (PMC12633147; doi:10.7717/peerj.20339)
Supplement: Supplemental Information 3 [file peerj-13-20339-s003.docx]

**Table S2:** Environmental parameters of the Southeastern Pacific Ocean: sea surface temperature, dissolved oxygen, salinity, and chlorophyll obtained from IMARPE, IFOP, and the web platforms Giovanni and Copernicus.

| **Latitude** | **Temperature (°C)** | **Dissolved Oxygen (mL/L)** | **Salinity (PSU)** | **Chlorophyll (mg/m^3^)** |
| --- | --- | --- | --- | --- |
|  | **Mean ± SD** | | | |
| **8°S** | 19.56 ± 1.01 | 0.42 ± 1.09 | 34.89 ± 0.10 | 7.47 ± 5.56 |
| **9°S** | 20.52 ± 1.50 | 0.53 ± 1.35 | 34.91 ± 0.11 | 7.42 ± 3.92 |
| **10°S** | 19.81 ± 1.99 | 1.40 ± 1.70 | 35.05 ± 0.05 | 6.83 ± 3.56 |
| **11°S** | 20.33 ± 2.38 | 1.68 ± 1.82 | 35.05 ± 0.07 | 5.86 ± 4.62 |
| **12°S** | 20.57 ± 1.91 | 0.65 ± 1.46 | 34.84 ± 0.13 | 5.00 ± 3.07 |
| **13°S** | 19.15 ± 1.89 | 0.45 ± 1.15 | 34.86 ± 0.14 | 2.75 ± 1.74 |
| **14°S** | 19.24 ± 1.34 | 0.92 ± 1.77 | 34.79 ± 0.12 | 2.75 ± 1.74 |
| **15°S** | 19.48 ± 1.48 | 0.48 ± 1.45 | 34.82 ± 0.11 | 2.91 ± 1.91 |
| **16°S** | 21.43 ± 1.42 | 0.83 ± 1.61 | 34.77 ± 0.12 | 1.80 ± 1.11 |
| **17°S** | 23.39 ± 1.66 | 0.69 ± 1.51 | 34.79 ± 0.08 | 0.78 ± 0.43 |
| **18°S** | 22.88 ± 1.57 | 0.48 ± 1.25 | 34.76 ± 0.10 | 1.65 ± 1.23 |
| **19°S** | 23.40 ± 1.28 | 3.20 ± 2.13 | 34.82 ± 0.12 | 0.39 ± 0.11 |
| **20°S** | 22.92 ± 1.36 | 3.58 ± 1.93 | 34.96 ± 0.11 | 0.32 ± 0.16 |
| **21°S** | 22.30 ± 1.39 | 3.40 ± 1.81 | 34.71 ± 0.10 | 1.11 ± 0.70 |
| **22°S** | 22.86 ± 1.10 | 4.55 ± 1.23 | 34.77 ± 0.11 | 0.31 ± 0.11 |
| **23°S** | 22.29 ± 1.42 | 3.70 ± 1.69 | 34.71 ± 0.09 | 0.26 ± 0.09 |
| **24°S** | 21.45 ± 1.57 | 4.35 ± 1.36 | 34.67 ± 0.06 | 0.55 ± 0.39 |
| **25°S** | 21.64 ± 1.32 | 4.75 ± 1.15 | 34.59 ± 0.07 | 0.28 ± 0.10 |
| **26°S** | 20.41 ± 1.21 | 4.95 ± 0.89 | 34.59 ± 0.04 | 0.62 ± 0.31 |
| **27°S** | 20.08 ± 1.26 | 4.51 ± 1.28 | 34.57 ± 0.04 | 0.62 ± 0.60 |
| **28°S** | 19.42 ± 1.39 | 4.54 ± 1.23 | 34.50 ± 0.05 | 0.43 ± 0.28 |
| **29°S** | 18.91 ± 1.25 | 4.08 ± 1.40 | 34.49 ± 0.04 | 0.66 ± 0.32 |
| **30°S** | 17.96 ± 1.29 | 4.33 ± 1.40 | 34.46 ± 0.05 | 0.95 ± 0.47 |
| **31°S** | 17.48 ± 1.04 | 4.07 ± 1.57 | 34.36 ± 0.05 | 1.47 ± 1.80 |
| **32°S** | 18.02 ± 0.71 | 4.08 ± 1.43 | 34.30 ± 0.06 | 1.42 ± 1.48 |
| **33°S** | 17.56 ± 0.94 | 4.09 ± 1.38 | 34.28 ± 0.06 | 2.02 ± 3.36 |
| **34°S** | 16.63 ± 0.88 | 3.30 ± 1.52 | 34.30 ± 0.12 | 0.82 ± 0.39 |
| **35°S** | 15.70 ± 0.72 | 2.37 ± 1.58 | 34.42 ± 0.16 | 1.81 ± 1.51 |
| **36°S** | 14.91 ± 0.61 | 2.01 ± 1.61 | 34.37 ± 0.27 | 3.28 ± 2.15 |
| **37°S** | 15.11 ± 0.98 | 3.67 ± 1.88 | 33.99 ± 0.23 | 1.28 ± 0.68 |
