# Supplementary material for: Latitudinal variations in morphometric traits and bioenergetic status of adult red squat lobsters Grimothea monodon (H. Milne Edwards, 1837) in the Southeast Pacific Ocean
Source: PeerJ. 2025 Nov 17;13:e20339. doi: 10.7717/peerj.20339 (PMC12633147; doi:10.7717/peerj.20339)
Supplement: Supplemental Information 4 — Note: Av.Abund, Average abundance; Av.Sim, Average similarity; Contrib %, Contribution Percentage; Cum %, Cummulative Contribution Percentage [file peerj-13-20339-s004.docx]

**Table S3:** Analysis of the percentage of similarity (SIMPER) of *Grimothea monodon* individuals: “small- pelagic (SP)” (09°S-17°S) and “large-benthic (LB)” (30°S-36°S) captured in the Southeastern Pacific Ocean

| **Trait** | **Sampling area** | **Average similarity** | **Fatty acid** | **Av.Abund** | **Av.Sim** | **Sim/SD** | **Contrib%** | **Cum%** |
| --- | --- | --- | --- | --- | --- | --- | --- | --- |
| **Small Pelagic** | **Chimbote (09°S)** | **89.24** | **C16:0** | 2.51 | 21.36 | 11.23 | 23.93 | 23.93 |
|  |  |  | **C18:1n9** | 2.14 | 18.02 | 9.93 | 20.2 | 44.13 |
|  |  |  | **C18:0** | 1.78 | 14.71 | 6.81 | 16.48 | 60.62 |
|  |  |  | **C14:0** | 1.71 | 14.28 | 11.29 | 16.01 | 76.62 |
|  |  |  | **C16:1** | 1.8 | 14.01 | 2.73 | 15.7 | 92.32 |
|  | **Huarmey (10°S)** | **83.63** | **C16:0** | 2.47 | 19.48 | 7.3 | 23.29 | 23.29 |
|  |  |  | **C18:1n9** | 2.06 | 15.05 | 3.21 | 18 | 41.29 |
|  |  |  | **C18:0** | 1.84 | 13.92 | 5.55 | 16.64 | 57.94 |
|  |  |  | **C14:0** | 1.62 | 12.19 | 6.24 | 14.57 | 72.51 |
|  |  |  | **C16:1** | 1.67 | 12 | 3.22 | 14.35 | 86.86 |
|  | **Huacho (11°S)** | **85.31** | **C16:0** | 2.61 | 22.29 | 9.76 | 26.13 | 26.13 |
|  |  |  | **C18:0** | 1.85 | 14.83 | 3.34 | 17.39 | 43.51 |
|  |  |  | **C14:0** | 1.63 | 13.58 | 11.3 | 15.92 | 59.44 |
|  |  |  | **C18:1n9** | 1.71 | 11.86 | 1.69 | 13.9 | 73.34 |
|  | **Lima (12°S)** | **80.91** | **C16:0** | 2.43 | 20.17 | 5.78 | 24.93 | 24.93 |
|  |  |  | **C18:1n9** | 2.04 | 15.54 | 2.63 | 19.2 | 44.13 |
|  |  |  | **C18:0** | 1.8 | 14.83 | 5.7 | 18.33 | 62.46 |
|  |  |  | **C14:0** | 1.77 | 14.62 | 5.94 | 18.07 | 80.52 |
|  |  |  | **C16:1** | 1.38 | 8.25 | 1.2 | 10.19 | 90.72 |
|  | **Cañete (13°S)** | **84.94** | **C16:0** | 2.57 | 21.17 | 6.51 | 24.92 | 24.92 |
|  |  |  | **C14:0** | 1.8 | 14.8 | 6.43 | 17.42 | 42.35 |
|  |  |  | **C18:0** | 1.76 | 14.15 | 5.64 | 16.65 | 59 |
|  |  |  | **C18:1n9** | 1.92 | 14.12 | 2.45 | 16.62 | 75.62 |
|  |  |  | **C16:1** | 1.51 | 10.27 | 1.96 | 12.09 | 87.72 |
|  | **Lomitas (14°S)** | **90.81** | **C16:0** | 2.5 | 18.7 | 11.04 | 20.6 | 20.6 |
|  |  |  | **C18:1n9** | 2.1 | 15.54 | 12.44 | 17.12 | 37.72 |
|  |  |  | **C18:0** | 1.85 | 13.43 | 7.85 | 14.79 | 52.51 |
|  |  |  | **C14:0** | 1.63 | 11.96 | 11.44 | 13.17 | 65.68 |
|  |  |  | **C16:1** | 1.49 | 9.94 | 2.54 | 10.95 | 76.63 |
|  | **Marcona (15°S)** | **92.44** | **C16:0** | 2.52 | 18.48 | 10.87 | 19.99 | 19.99 |
|  |  |  | **C18:1n9** | 2.07 | 14.44 | 3.44 | 15.62 | 35.61 |
|  |  |  | **C18:0** | 1.76 | 12.76 | 11.68 | 13.81 | 49.41 |
|  |  |  | **C14:0** | 1.66 | 12 | 9.75 | 12.98 | 62.39 |
|  |  |  | **C16:1** | 1.63 | 11.79 | 18.44 | 12.76 | 75.15 |
|  | **Chala (16°S)** | **88.42** | **C16:0** | 2.53 | 19.62 | 8.37 | 22.18 | 22.18 |
|  |  |  | **C18:1n9** | 1.99 | 14.39 | 2.8 | 16.27 | 38.46 |
|  |  |  | **C18:0** | 1.79 | 13.65 | 7.49 | 15.44 | 53.89 |
|  |  |  | **C14:0** | 1.65 | 12.75 | 8.85 | 14.42 | 68.31 |
|  |  |  | **C16:1** | 1.61 | 11.71 | 4 | 13.24 | 81.55 |
|  | **Planchada (16°34'S)** | **87.2** | **C16:0** | 2.55 | 19.7 | 8.68 | 22.59 | 22.59 |
|  |  |  | **C18:1n9** | 1.97 | 14.45 | 3.32 | 16.57 | 39.16 |
|  |  |  | **C14:0** | 1.71 | 13 | 8.15 | 14.9 | 54.06 |
|  |  |  | **C18:0** | 1.7 | 12.9 | 8.13 | 14.8 | 68.86 |
|  |  |  | **C16:1** | 1.66 | 12.59 | 8.96 | 14.43 | 83.29 |
|  | **Mollendo (17°S)** | **87.11** | **C16:0** | 2.53 | 19.73 | 7.18 | 22.64 | 22.64 |
|  |  |  | **C18:1n9** | 2 | 14.42 | 2.76 | 16.55 | 39.2 |
|  |  |  | **C14:0** | 1.75 | 13.67 | 7.56 | 15.7 | 54.89 |
|  |  |  | **C16:1** | 1.75 | 12.67 | 3.13 | 14.54 | 69.43 |
|  |  |  | **C18:0** | 1.62 | 12.46 | 7.23 | 14.31 | 83.74 |
| **Large Benthic** | **Coquimbo (33°S)** | **80.52** | **C16:0** | 2.35 | 16.94 | 6.11 | 21.04 | 21.04 |
|  |  |  | **C18:1n9** | 1.97 | 14 | 7.13 | 17.39 | 38.42 |
|  |  |  | **C18:0** | 1.84 | 13.22 | 5.87 | 16.41 | 54.84 |
|  |  |  | **C14:0** | 1.55 | 11 | 5.65 | 13.66 | 68.5 |
|  |  |  | **C17:0** | 1.5 | 10.82 | 6.15 | 13.44 | 81.94 |
|  | **Concepción (36°S)** | **90.01** | **C16:0** | 2.13 | 12.95 | 7.86 | 14.39 | 14.39 |
|  |  |  | **C18:1n9** | 2.04 | 12.67 | 14.57 | 14.08 | 28.47 |
|  |  |  | **C20:5n3** | 1.87 | 11.16 | 7.89 | 12.4 | 40.87 |
|  |  |  | **C18:0** | 1.79 | 11.03 | 14.44 | 12.25 | 53.12 |
|  |  |  | **C22:6n3** | 1.79 | 10.85 | 10.48 | 12.06 | 65.18 |

Av.Abund = Average abundance, Av.Sim = Average similarity, Contrib % = Contribution Percentage, Cum % = Cummulative Contribution Percentage
