# Supplementary material for: Latitudinal variations in morphometric traits and bioenergetic status of adult red squat lobsters Grimothea monodon (H. Milne Edwards, 1837) in the Southeast Pacific Ocean
Source: PeerJ. 2025 Nov 17;13:e20339. doi: 10.7717/peerj.20339 (PMC12633147; doi:10.7717/peerj.20339)
Supplement: Supplemental Information 5 [file peerj-13-20339-s005.docx]

**Table S4:** Analysis of similarity (ANOSIM) of *Grimothea monodon* individuals: “small- pelagic (SP)” (09°S-17°S) and “large-benthic (LB)” (30°S-36°S) captured in the Southeastern Pacific Ocean.

|  | **Chimbote (09°S)** | **Huarmey (10°S)** | **Huacho (11°S)** | **Lima (12°S)** | **Cañete (13°S)** | **Lomitas (14°S)** | **Marcona (15°S)** | **Chala (16°S)** | **Planchada (16°34’S)** | **Mollendo (17°S)** | **Coquimbo (33°S)** | **Concepción (36°S)** |
| --- | --- | --- | --- | --- | --- | --- | --- | --- | --- | --- | --- | --- |
| **Chimbote (09°S)** |  |  |  |  |  |  |  |  |  |  |  |  |
| **Huarmey (10°S)** | 0.38 |  |  |  |  |  |  |  |  |  |  |  |
| **Huacho (11°S)** | 0.25 | 0.14 |  |  |  |  |  |  |  |  |  |  |
| **Lima (12°S)** | 0.44 | 0.13 | 0.42 |  |  |  |  |  |  |  |  |  |
| **Cañete (13°S)** | 0.69 | 0.34 | 0.58 | 0.02 |  |  |  |  |  |  |  |  |
| **Lomitas (14°S)** | 0.86 | 0.42 | 0.63 | 0.2 | 0.17 |  |  |  |  |  |  |  |
| **Marcona (15°S)** | 0.88 | 0.37 | 0.61 | 0.12 | 0.15 | 0.02 |  |  |  |  |  |  |
| **Chala (16°S)** | 0.77 | 0.39 | 0.56 | 0.16 | 0.16 | 0.12 | 0.2 |  |  |  |  |  |
| **Planchada (16°34’S)** | 0.68 | 0.27 | 0.57 | 0.07 | 0.02 | 0.13 | 0.15 | 0.13 |  |  |  |  |
| **Mollendo (17°S)** | 0.76 | 0.36 | 0.58 | 0.1 | 0.28 | 0.29 | 0.26 | 0.38 | 0.23 |  |  |  |
| **Coquimbo (33°S)** | 0.96 | 0.8 | 0.73 | 0.53 | 0.72 | 0.56 | 0.54 | 0.73 | 0.69 | 0.64 |  |  |
| **Concepción (36°S)** | 0.99 | 0.85 | 0.91 | 0.5 | 0.69 | 0.57 | 0.6 | 0.68 | 0.62 | 0.82 | 0.37 |  |
